# A temporal assessment of nematode community structure and diversity in the rhizosphere of cisgenic *Phytophthora infestans*-resistant potatoes

**DOI:** 10.1186/s12898-016-0109-5

**Published:** 2016-12-01

**Authors:** Vilma Ortiz, Sinead Phelan, Ewen Mullins

**Affiliations:** 1 Dept. Crop Science, Teagasc, Oak Park, Carlow, Ireland; 2 Plant Biology and Crop Science, Rothamsted Research Station, West Common, Harpenden, Hertfordshire AL5 2JQ UK

**Keywords:** GM cisgenic potato, Nematode, Diversity, Community structure

## Abstract

**Background:**

Nematodes play a key role in soil processes with alterations in the nematode community structure having the potential to considerably influence ecosystem functioning. As a result fluctuations in nematode diversity and/or community structure can be gauged as a ‘barometer’ of a soil’s functional biodiversity. However, a deficit exists in regards to baseline knowledge and on the impact of specific GM crops on soil nematode populations and in particular in regard to the impact of GM potatoes on the diversity of nematode populations in the rhizosphere. The goal of this project was to begin to address this knowledge gap in regards to a GM potato line, cisgenically engineered for resistance to *Phytophthora infestans* (responsible organism of the Irish potato famine causing late blight disease). For this, a 3 year (2013, 2014, 2015) field experimental study was completed, containing two conventional genotypes (cvs. Desiree and Sarpo Mira) and a cisgenic genotype (cv. Desiree + *Rpi*-*vnt1*). Each potato genotype was treated with different disease management strategies (weekly chemical applications and corresponding no spray control). Hence affording the opportunity to investigate the temporal impact of potato genotype, disease management strategy (and their interaction) on the potato rhizosphere nematode community.

**Results:**

Nematode structure and diversity were measured through established indices, accounts and taxonomy with factors recording a significant effect limited to the climatic conditions across the three seasons of the study and chemical applications associated with the selected disease management strategy. Based on the metrics studied, the cultivation of the cisgenic potato genotype exerted no significant effect (P > 0.05) on nematode community diversity or structure. The disease management treatments led to a reduction of specific trophic groups (e.g. Predacious c–p = 4), which of interest appeared to be counteracted by a potato genotype with vigorous growth phenotype e.g. cv. Sarpo Mira. The fluctuating climates led to disparate conditions, with enrichment conditions (bacterial feeding c–p = 1) dominating during the wet seasons of 2014 and 2015 versus the dry season of 2013 which induced an environmental stress (functional guild c–p = 2) on nematode communities.

**Conclusions:**

Overall the functional guild indices in comparison to other indices or absolutes values, delivered the most accurate quantitative measurement with which to determine the occurrence of a specific disturbance relative to the cultivation of the studied cisgenic *P. infestans*-resistant potatoes.

**Electronic supplementary material:**

The online version of this article (doi:10.1186/s12898-016-0109-5) contains supplementary material, which is available to authorized users.

## Background

In terms of global production potato (*Solanum tuberosum* L.) is the fourth most important global food crop after, maize, wheat and rice [1]. Yet, the same crop that sustains human dietary requirements across the world is susceptible to a myriad of diseases; the most economically significant [2] being potato late blight disease (causative organism *Phytophthora infestans*), which continues to ‘emerge’ with devastating affect [3]. While chemical control measures have maintained yields, European regulations on the use of plant protection products [4] present an additional challenge to commercial potato growers at a time when novel, more aggressive strains of *P. infestans* are dominating native populations [5]. Looking ahead, the deployment of genetic resistance into commercial varieties is the only logical solution [6, 7], in light of the legislative and environmental challenges facing the crop [8].

However, the introgression of resistance (R) genes from wild potato species into breeding populations via conventional practise is a time consuming and logistically challenging process [9], which is further complicated by the evolving potential of *P. infestans* to adapt and overcome R genes [10, 11]. However, as the characterisation of R genes has rapidly increased [7, 9, 12] in parallel to the mainstream adoption of sequencing technologies, the concept of stacking R genes via cisgenic genetic modification to deliver durable resistance is now a reality [13]. This theory is more sustainable if merged with an appropriate Integrated Pest Management (IPM) strategy [2], with the agronomic potential of a suite of R genes having been recently demonstrated in field evaluations in Belgium [14], the Netherlands [15] and separately in the UK [16].

From the perspective of the European legal framework [17], genetically modified (GM) crops must undergo a comprehensive risk assessment prior to market release; the goal of which is to determine the level of substantial equivalence between the engineered material and its conventional comparator in regards to human and animal health and the environment. Coordinated by the European Food Safety Authority (EFSA), these assessments are supported by the EFSA GMO panel, which in 2010 proposed a novel risk assessment approach for European environments based on the selection of functional groups and/or individual species within a tiered approach, such that the focus is on the analysis of functional biodiversity in receiving environments and the possible interference GM varieties could cause to the functioning of this habitat [18]. To accomplish this though, risk assessment investigations require scientific data about the possible environmental impact of cultivating a GM variety and to achieve this, a higher level of practical research is required that relates directly to the field environment [19]. An important component of this is the overall impact cultivation may have on soil biodiversity, which supports a diversity of microbes (fungi, bacteria and algae), microfauna (protozoa) and mesofauna organisms such as arthropods and nematodes, all of which are critical to soil functionality.

Nematodes are key agents in important soil processes such as decomposition, mineralisation and nutrient cycling, with alterations in the nematode community structure having the potential to considerably influence ecosystem functioning [20]. Widespread and highly diverse, nematodes form part of the food web of soil by occupying primary, secondary and tertiary positions in at least five trophic groups: bacterial feeding (BF), fungal feeding (FF), predators (PR), omnivorous (OM) and plant feeding (PF) [21], making them excellent indicators of fluctuations in soil composition arising from for example, plant genotype and/or type of soil management and environmental conditions in the rhizosphere. To date, multiple studies have been carried out using soil nematodes as indicators in different ecosystems evaluating for example the impact of crop management [22, 23], fertilizers [24], water availability [25], seasonal fluctuations [26] as well as the application of crop protectants [27]. From the perspective of monitoring nematode diversity in response to the cultivation of GM crops, several reports have detailed interactions in regards to GM maize, carrying Cry-type insecticidal proteins [28, 29]. However, to date no study has detailed the impact on nematode diversity of cultivating GM potato. This issue is compounded by the fact that a knowledge deficit also exists in regards to describing nematode community diversity within the rhizosphere of cultivated potatoes as a whole.

The process of characterizing nematode populations can be achieved morphologically or via the sequencing of nuclear (LSU rDNA, SSU rDNA and ITS) and/or mitochondrial genes (Cytochrome c oxidase subunit). Of the targets listed the SSU rDNA has proven to be most informative for investigating nematode populations considering the semi-conserved and variable regions within the sequence which provides opportunity to identify down to the species level [30]. From this, taxonomic conclusions along with absolute values and respective indices, that integrate the responses of different nematode taxa and trophic groups to soil perturbations, can be calculated as a means to measure environmental impact on the soil ecosystem [31, 32]. In light of the application of high-throughput sequencing for characterising nematode communities, balancing the desire to achieve adequate coverage of samples taken versus the cost of detecting sequences within same samples is an important consideration. While Neher and Campbell [33] examined the issue of optimal sampling strategies via the variability of ecological indices, richness and evenness indices could also be alternative parameters with which to determine the level of inter-replicate variability.

In providing a framework for quantifying the environmental impact of a GM crop, the EFSA Guidance Document [18] details a number of areas that require focus, including; impacts of GM crops on soil biodiversity and biology. The goal of this study was to begin the process of generating a baseline, from which the temporal impact (2013–2015) of cultivating modified cisgenic potatoes (equipped with a single R gene derived from *Solanum venturii*) on soil nematode community structure and diversity could begin to be quantified. As a comparative study, the work also included the opportunity to calculate nematode diversity relative to conventional potato practises that rely on weekly chemical fungicide applications and the cultivation of an additional potato cultivar Sarpo Mira, generated through conventional breeding but which possesses five sources of genetic resistance [9]. Combined, this work provides insight into the overall impact of this specific cisgenic potato crop on soil nematode populations and begins to address the current knowledge deficit that exists in the literature on this subject. Completed as part of the EU funded AMIGA project (http://www.amigaproject.eu), the output of this study contributes to the overall AMIGA goal of supporting policymakers and society in developing an in depth understanding of the potential impacts associated with the field cultivation of GM crops in the EU [19].

## Methods

### Weather measurements

Information on the rainfall (mm), environmental temperature (°C), % relative humidity and soil temperature (°C) at a 30 cm depth were recorded daily at the Oak Park automated weather station, situated ~400 m from the field site but which is linked with a national network of weather stations (http://www.met.ie). Rainfall and soil temperature were considered as direct parameters affecting the nematode community, while environmental temperature and relative humidity were considered indirect parameters involved in the cultivation of the potatoes.

### Experimental design, plant material and crop husbandry

The study was completed on the Oak Park campus of the Teagasc Crop Research Centre, Carlow, Ireland (GPS coordinates; 52.8560667, −6.9121167), where a fixed field (~1 ha) was split into two equal sites (No. 1 and No. 2). Previously a low-managed grass pasture for >10 years, for 2013, 2014 and 2015 each site was cultivated with plots of three potato genotypes; the conventional cultivar Desiree, the modified cisgenic Desiree line and the conventionally bred Sarpo Mira cultivar, with each genotype undergoing three treatments corresponding to a weekly chemical fungicide spray regime, a decision support system-based spray regime and a control ‘no spray’ treatment. For the purposes of this study only the weekly chemical (‘chemical’) and no spray (‘control’) treatments were examined. This led to six treatments in total being considered; Desiree control, Desiree chemical, cisgenic Desiree control, cisgenic Desiree chemical, Sarpo Mira control and Sarpo Mira chemical. The cisgenic line was previously engineered to contain a single copy of the *Rpi*-*vnt1.1* gene (derived from *S. venturii*), which confers resistance to the late blight pathogen *Phytophthora infestans* [34, 35] and was provided to the AMIGA project via the DuRPh programme (http://www.DuRPh.nl) of Wageningen University. Each genotype × treatment plot measured 3 m × 3 m with plots separated on all sides by 3 m of grass. Each site contained 54 plots randomised in order across 6 replicating blocks with 9 plots (3 genotypes × 3 treatments) per block. From year-to-year plots were rotated through the 1 ha site to ensure that for each year plots were only positioned on land that was original grass pasture. This strategy was important to minimise the accumulation of soil-borne potato diseases in the soil as a result of repeat cropping but also from the nematode perspective it ensured that the ‘starting point’ for each plots was the same each year; by sowing them on original grass pasture. Plots received the same crop management protocols (with the exception of chemical fungicide treatments) indistinct of the genotype evaluated. Sites were prepared by deep ploughing and rotavating before standard commercial potato drills were formed through each block of nine plots.

### Soil sampling and nematode extraction

A flowchart detailing the experiment design, genotypic characteristics of the three potato genotypes grown, soil sampling as well as sample preparation for molecular analysis is presented in Additional file 1: Figure S1. Soil samples were collected from the plant rhizosphere at the initiation of flowering, which was typically during the first 2 weeks of August of each year. For each of the 6 treatments, 7 plots were randomly selected (4 from site 1 and 3 from site 2) and within each of the seven plots (per treatment), one plant was selected and with soil still attached to the roots, carefully placed inside a bag for transfer to the laboratory. Upon arrival soil adhering to the roots was scraped into the same bag and the plant removed. The remaining soil in the bag was thoroughly mixed before 100 g was removed for placing in a labelled plastic bag which was sealed and stored at 4 °C. Nematodes were extracted by processing 100 g of the homogenized soil/plot (seven replicates/treatment) via an Oostenbrink elutriator, followed by passage through a series of sieves (45, 90, 125 and 180 mesh size) and then a cotton wool filter. After a 48 h incubation period at room temperature, a volume of 50 ml was then recovered from the cotton wool filter, from which nematodes were collected into 10 ml following a 4 °C treatment for 24 h. Final volumes were subsequently stored at −80 °C. Across the 3 years (2013, 2014, 2015) of the study a total of 126 soil samples were processed in this manner.

### DNA extraction

Each 10 ml sample was freeze dried overnight before DNA was extracted as per the Purelink Genomic DNA kit (Invitrogen/Cat No. 1820-01) with an adapted protocol for nematode DNA. Modifications included: 360 μl of Purelink Genomic buffer plus 40 μl of proteinase K was added to each tube which was then agitated at 55 °C overnight. The suspension was then centrifuged (13,000 rpm, 3 min) and the resulting supernatant processed as per kit’s recommendations, with the exception that the DNA was eluted from the column using 100 μl sterile water. All eluted samples were stored at −40 °C.

### Target sequence amplification and sequencing

The 5′ end of the 18 small subunit rDNA gene (~1000 bp) was amplified using a set of universal primers (SSU18A and SSU26R [36]). All PCR reactions were completed in a 50 μl volume containing 50 ng of DNA template, 5 μl of 10× PCR buffer, 1 μl of each primer (10 mM) and 200 μM dNTP, with cycling conditions of; 95 °C–5 min, 30 × (95 °C–30 s, 60 °C–60 s, 72 °C–5 min), 72 °C–10 min. Five individual PCR reactions were completed for each of the seven samples per treatment to ensure adequate generation of the target amplicon, after which the PCR reaction volumes for each set of seven samples (per treatment) was pooled to deliver a composite PCR sample for each treatment. Each composite sample was then cloned into *E. coli* (p-GEM, Promega) and 50 individual colonies (per treatment/year) were randomly selected and sent to an external provider for Sanger sequencing. Acquired sequences were analysed against the GenBank database using standard BLAST analysis with alignments and clustering completed with Clustal 1× and Mega. Owing to low DNA concentrations attained with some of the 2013 samples, a nested PCR approach was required and implemented with the secondary PCR (to that detailed previously) employing the SSU9R and S18 primers [36], which generate a nested fragment ~500 bp. All sequences were deposited in the NCBI GenBank under accession numbers: KY119383–KY119427, KY119428–KY119476, KY119477–KY119513, KY119514–KY119563, KY119564–KY119588, KY119589–KY119632, KY119633–KY119676, KY119677–KY119724, KY119725–KY119770, KY119771–KY119811, KY119812–KY119853, KY119854–KY119901, KY119902–KY119944, KY119945–KY119986, KY119987–KY120026, KY120027–KY120072, KY120073–KY120120, KY120121–KY120164.

### Nematode community analysis

To fully characterize the nematode community structure (NCS) in the respective rhizospheric samples, the NCS was analysed on the 18 composite samples in terms of indices, absolute values and qualitative taxonomy with both free living and plant parasitic groups evaluated. The indices analysis was divided into ecological measurement with the Maturity index (MI) (and its variant MIMO and ∑MIMO), in functional guild indices: enrichment index (EI), structure index (SI), chanel index (CH), bacterial feeding (BF) c–p = 1 and 2 and diversity indices: Shannon index (H), Shannon equitability (EH), Simpson index (D), Simpson index, probability of diversity (1-D) and the Simpson reciprocal index (1/D). The interaction structure (SI) and enrichment (EI) values were analysed through a graphical representation of the nematode faunal analysis and absolute values determining family, genus and species numbers. Sorensen coefficients for evaluating similarity between treatments were also calculated. A summary of all calculations realized is described in Additional file 2: Table S1. Finally, the taxonomy at family and genus level was evaluated to find possible bio-indicators of disturbance of the environment related to the different potato genotypes and chemical treatments applied.

### Data and statistical analysis

To verify that the number of clones extracted from the composite samples (sampling effort) would provide sufficient information (how well the community has been sampled) on the total nematode richness (species and genus) in the rhizosphere, an individual-based rarefaction analysis was completed using the R package “stat” [37]. To estimate the effect on the nematode community of potato genotype, chemical treatment and the interaction between the two, a two-way analysis of variance (ANOVA) was performed with data blocked per year. When effects were significant, multiple comparisons between the means were made as per the LSD test, with differences at probability of P ≤ 0.01 and P ≤ 0.05 considered. All analyses were performed using GenStat software v.18. To show in more detail the number of family, genus and species, identity of observed species and genus and trophic groups richness by each interaction genotype and disease management per year (2013–2015) a two dimensional representation (heatmap) was performed for each case using the GenStat software v.18.

## Results

### Climatic conditions for the 2013, 2014 and 2015 field studies

In contrast to the 1st (2013) and 3rd (2015) year of the study, 2014 was generally characterized by higher soil temperatures and relative humidity (Table 1; Additional file 3: Figure S2). However, focussing on months for cultivation only (May to August), air and soil temperature along with relative humidity varied significantly across all 3 years (Additional file 3: Figure S2). In relation to rainfall, in 2014, May and August were the months in which the most precipitation was registered and for 2015 it was May and July. The 2013 field season experienced scarce rainfall, with significantly lower values (P < 0.05) of relative humidity, compared to 2014 and 2015, but with higher air and soil temperatures for June to August and July to August respectively (Table 1).

**Table 1:** Comparative analysis of monthly rainfall (R), air temperature (AT), soil temperature (ST) and relative humidity (RH) measurements made from January to August for 2013, 2014 and 2015 at the field site in Oak Park, Carlow, Ireland

Month	Rainfall			Air temperature			Soil temperature			Relative humidity		
	F value	P value	Year^a^	F value	P value	Year^a^	F value	P value	Year^a^	F value	P value	Year^a^
January	3.09	<0.051	2014^b^	0	1		1.07	0.348		0	1	
February	16.9	<0.001	2014^b^	0.28	0.759		4.12	<0.02	2014	4.59	<0.013	2014^b^
March	0.09	0.912	2014	3.41	<0.038	2013^b^, 2014	29.85	<0.001	2014^b^, 2015	5.87	<0.004	2014^b^
April	0.85	0.429	2014	17.52	<0.001	2014^b^, 2015	30.69	<0.001	2014^b^, 2015	8.88	<0.001	2014^b^
May	2.21	0.115	2015	5.44	<0.006	2014^b^, 2015	4.95	<0.009	2014^b^	6.86	<0.002	2014^b^
June	0.83	0.439	2014	65.25	<0.001	2013^b^, 2015	4.5	<0.014	2014^b^	9.74	<0.001	2014^b^, 2015
July	3.12	<0.049	2015^b^	2.42	0.095	2013	45.07	<0.001	2013^b^, 2014	5.86	<0.004	2014^b^
August	0.30	0.742	2014	28.23	<0.001	2013^b^	22.03	<0.001	2013^b^	5.25	<0.007	2015^b^

^a^Years with the highest mean monthly value for the respective month and metric

^b^Respective year in which recorded month differed significantly (P < 0.05) from same month in other 2 years

### Rarefaction analysis

**Fig. 1:**
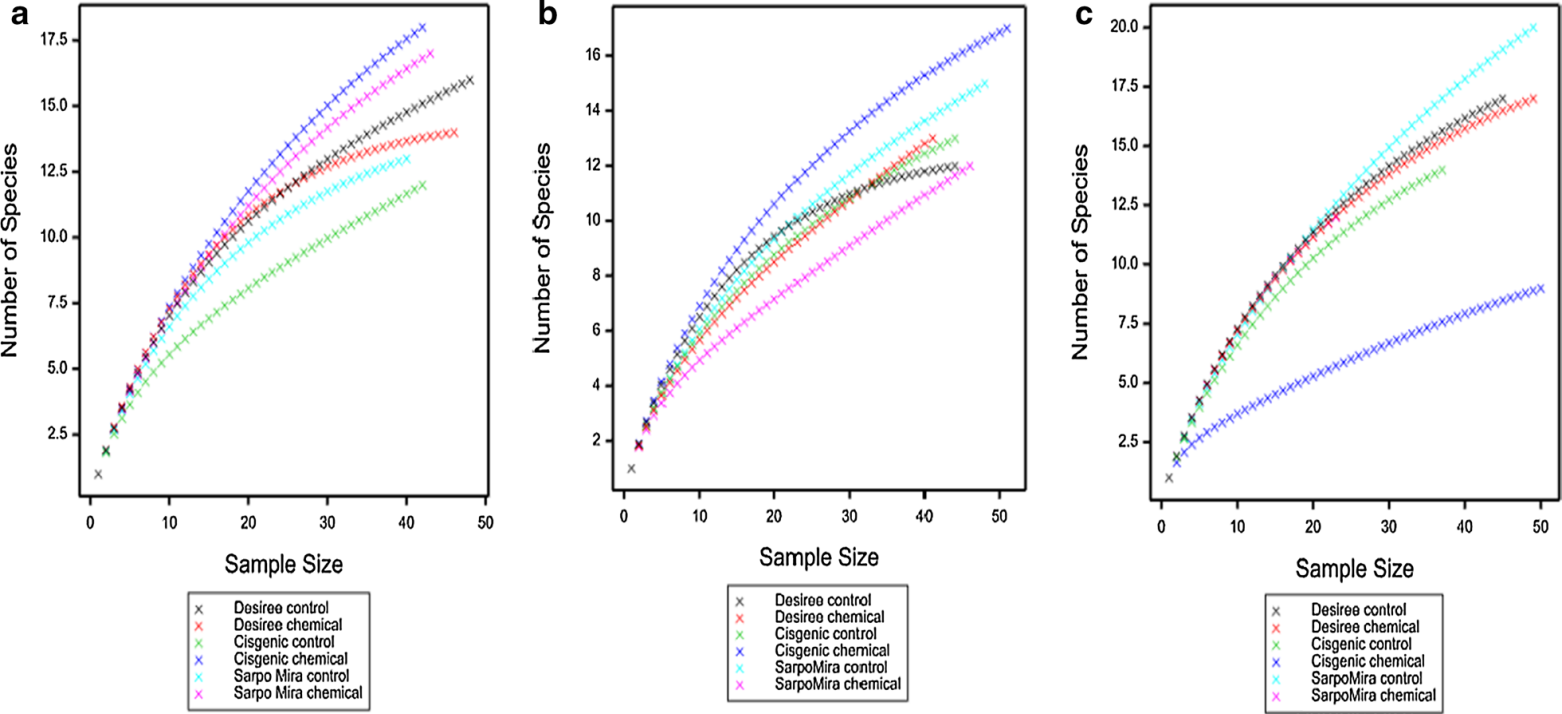
Rarefaction curves indicating the numbers of species observed relative to the number of clones sequenced, taken from the rhizosphere of potatoes genotypes (Desiree, cisgenic Desiree and Sarpo Mira) cultivated at Oak Park (Carlow, Ireland) under two different disease management regimes (control and chemical treatment) through 2013 (**a**), 2014 (**b**) and 2015 (**c**)

**Fig. 2:**
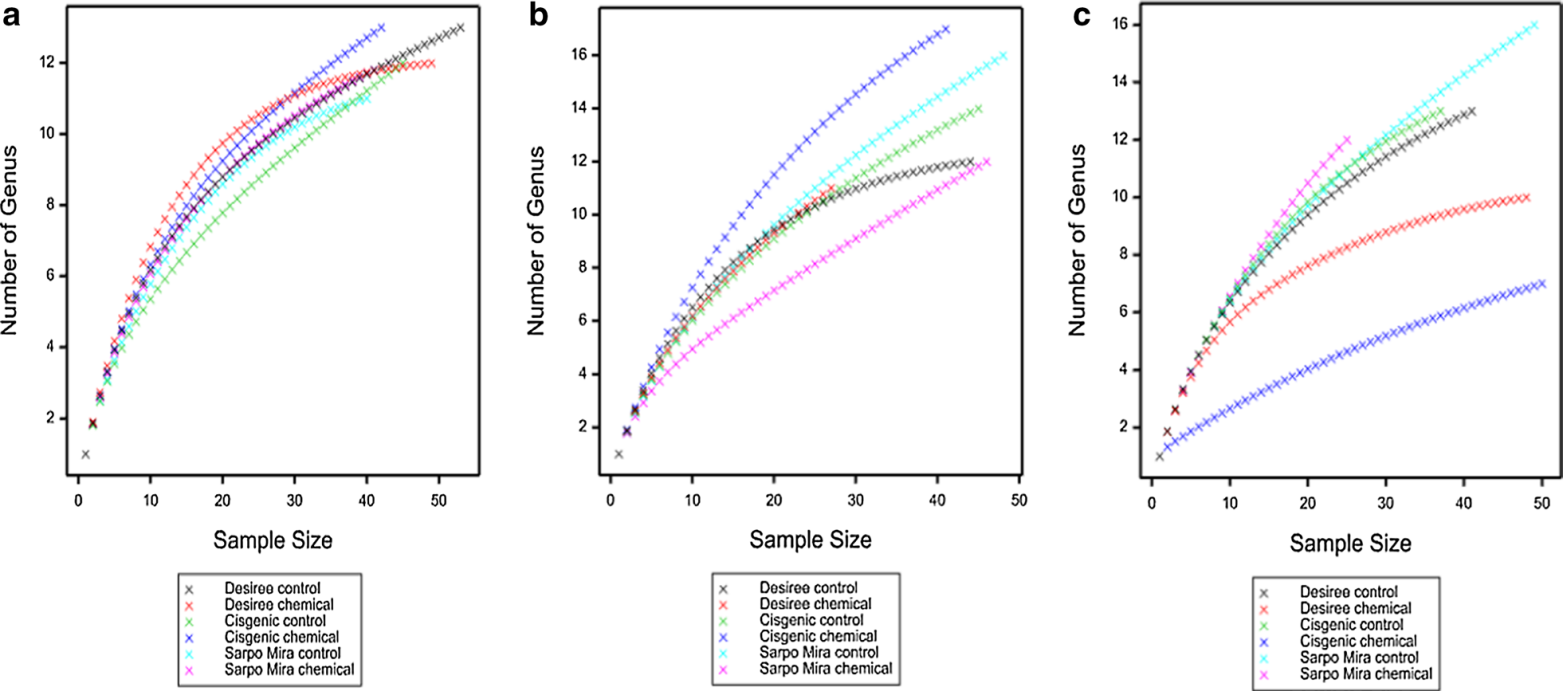
Rarefaction curves indicating the numbers of genus observed relative to the number of clones sequenced, taken from the rhizosphere of potatoes genotypes (Desiree, cisgenic Desiree and Sarpo Mira) cultivated at Oak Park (Carlow, Ireland) under two different disease management regimes (control and chemical treatment) through 2013 (**a**), 2014 (**b**) and 2015 (**c**)

The rarefaction analysis, which was completed for both nematode species (Fig. 1) and genus (Fig. 2) for all treatments over the 3 years of the study, illustrated the levels of nematode richness identified through the adopted strategy. While the chemical treated cisgenic potato samples returned the highest genus and species richness through 2013 and 2014 this was not the case in the final year of the study, 2015. Across the six treatments studied, the cv. Desiree derived samples recorded the lowest degree of fluctuation between the two disease management strategies applied, in contrast to cv. Sarpo Mira and cisgenic Desiree.

### Ecological succession indices

Mean maturity index (MI) values (Table 2) obtained for samples taken from untreated plots were higher (2.36 for Desiree; 2.05 for cisgenic Desiree; 2.13 for Sarpo Mira) than those recorded in the presence of the chemical fungicide treatment (1.93 for Desiree; 1.69 for cisgenic Desiree; 1.91 for Sarpo Mira). For the plant parasitic index (PPI) a similar trend (for control vs. weekly chemical treatment) was observed for cisgenic Desiree and Desiree only (2.83 vs. 1.10, Desiree; 2.63 vs. 1.83, cisgenic Desiree) and again with the PPI/MI (1.28 vs. 0.53, Desiree; 1.28 vs. 0.95, cisgenic Desiree) the modified maturity index (MIMO) (2.68 vs. 2.54, Desiree; 2.42 vs. 2.38, cisgenic Desiree) and the ∑MIMO (2.80 vs. 2.68, Desiree; 2.46 vs. 2.54 cisgenic Desiree). In contrast, a converse trend was noted with Sarpo Mira (Table 2).

**Table 2:** Impact of potato genotype (Desiree, cisgenic Desiree, Sarpo Mira), disease management (control, chemical treatment) and year (2013, 2014, 2105) on nematode community ecological succession indices [maturity index (MI), plant parasite index (PPI), modified MI to include removing of the c–p = 1 family (MIMO) and removing of the c–p = 1 family but with inclusion of the PPI to generate ∑MIMO] from rhizospheric samples taken from Oak Park field site

Index	Potato genotype	Disease management	2015	2014	2013	Mean	Mean/genotype	Mean/disease management
MI	Desiree	Control	3.05	1.76	2.27	2.36	2.14	2.18
	Desiree	Chemical	2.10	1.61	2.07	1.93		1.84
	Cisgenic Desiree	Control	2.03	2.15	1.97	2.05	1.87	
	Cisgenic Desiree	Chemical	1.16	2.13	1.79	1.69		
	Sarpo Mira	Control	2.63	1.76	2.00	2.13	2.02	
	Sarpo Mira	Chemical	1.86	1.56	2.30	1.91		
	Mean		2.14	1.83	2.07			
PPI	Desiree	Control	3.00	3.50	2.00	2.83	1.97	2.70
	Desiree	Chemical	0.00	0.00	3.31	1.10		2.10
	Cisgenic Desiree	Control	2.50	3.00	2.40	2.63	2.23	
	Cisgenic Desiree	Chemical	0.00	2.67	2.83	1.83		
	Sarpo Mira	Control	3.00	2.71	2.29	2.67	3.01	
	Sarpo Mira	Chemical	3.00	3.67	3.39	3.35		
	Mean		1.92	2.59	2.70			
PPI/MI	Desiree	Control	0.98	1.99	0.88	1.28	0.91	1.28
	Desiree	Chemical	0.00	0.00	1.60	0.53		1.10
	Cisgenic Desiree	Control	1.23	1.40	1.22	1.28	1.11	
	Cisgenic Desiree	Chemical	0.00	1.25	1.58	0.95		
	Sarpo Mira	Control	1.14	1.54	1.15	1.28	1.54	
	Sarpo Mira	Chemical	1.61	2.35	1.47	1.81		
	Mean		0.83	1.42	1.32			
MIMO	Desiree	Control	3.46	2.47	2.10	2.68	2.61	2.53
	Desiree	Chemical	3.00	2.39	2.23	2.54		2.57
	Cisgenic Desiree	Control	2.33	*2.94*	2.00	2.42	2.40	
	Cisgenic Desiree	Chemical	2.33	*2.80*	2.00	2.38		
	Sarpo Mira	Control	2.83	2.63	2.00	2.49	2.64	
	Sarpo Mira	Chemical	3.38	2.71	2.30	2.80		
	Mean		2.89	2.66	2.11**			
∑MIMO	Desiree	Control	3.42	2.89	2.08	2.80	2.74	2.61
	Desiree	Chemical	3.00	2.39	2.64	2.68		2.74
	Cisgenic Desiree	Control	2.34	2.94	2.09	2.46	2.50	
	Cisgenic Desiree	Chemical	2.33	2.79	2.50	2.54		
	Sarpo Mira	Control	2.84	2.68	2.13	2.55	2.79	
	Sarpo Mira	Chemical	3.30	2.88	2.88	3.02		
	Mean		2.87	2.76	2.39			

* P < 0.05, ** P < 0.01, *** P < 0.001

Considering crop genotype as an individual factor, cisgenic Desiree derived samples recorded the lowest mean values for the MI (1.87), MIMO (2.40) and ∑MIMO (2.50) indices compared to its direct comparator cv. Desiree and the alternative conventionally bred variety Sarpo Mira, which obtained the higher mean values for the PPI (3.01), PPI/MI (1.54), MIMO (2.64) and ∑MIMO (2.79) indices. When ranked (lowest to highest) for the MIMO and ∑MIMO indices, genotypes ordered as cisgenic Desiree, Desiree, Sarpo Mira whereas for the PPI and PPI/MI indices genotypes ranked as Desiree, cisgenic Desiree, Sarpo Mira. In the case of the MI index, mean values delivered an ordered ranking of cisgenic Desiree, Sarpo Mira, Desiree. Examining the impact of disease management (independent of the potato genotype sown), the means values for MI (2.18/1.84), PPI (2.70/2.10) and PPI/MI (1.28/1.10) proportions were larger in the absence of the chemical fungicide treatment (Table 2). The opposite was noted for MIMO (2.53/2.57) and ∑MIMO (2.61/2.74).

Statistically, weak effects were noted for the effect of chemical treatment on the MI index (P < 0.13), as well as the interaction of genotype × chemical treatment on the PPI (P < 0.20) and the PPI/MI ratio (P ≤ 0.18). The year sampled had a weak effect on ∑MIMO (P < 0.08) while in the case of the MIMO index 2013 differed significantly from 2014 to 2015 (P < 0.01, Table 2).

### Trophic groups

**Fig. 3:**
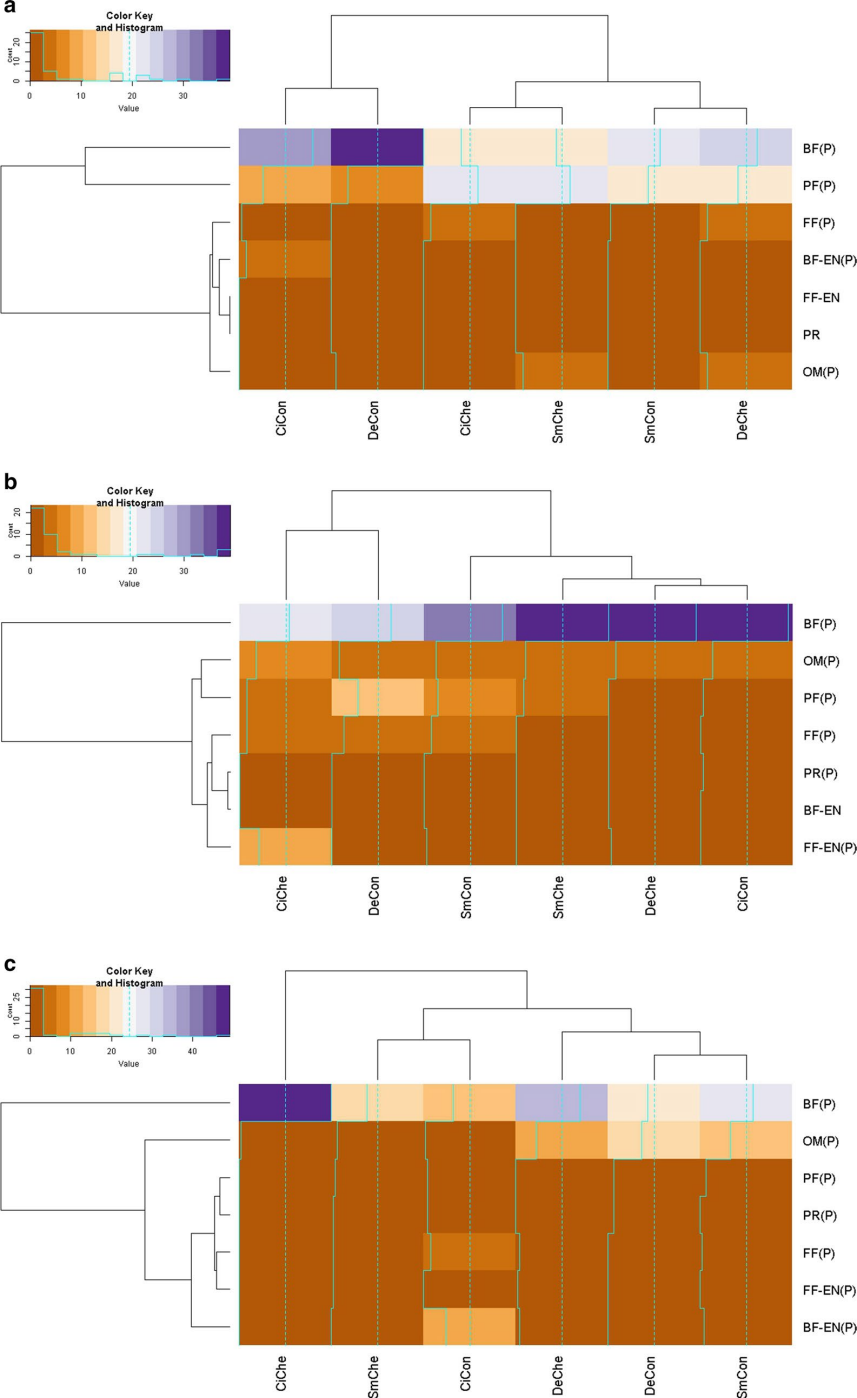
Heatmaps depicting impact of genotype (Desiree, cisgenic Desiree and Sarpo Mira), disease management (control, chemical) and year (2013, **a**; 2014, **b**; 2015, **c**) on the prevalence of nematodes from trophic groups representing bacterial feeding (BF), plant feeding (PF), fungal feeding (FF), omnivorous (OM), predacious (PR), fungal feeding or entomopathogens (FF or EN) and bacterial feeding or entomopathogens (BF or EN). Genotype × management interactions are labelled as: Desiree control [DeCon], Desiree chemical [DeChe], cisgenic Desiree control [CiCon], cisgenic Desiree chemical [CiChe], Sarpo Mira control [SmCon], Sarpo Mira chemical [SmChe]. (P) = present. For the colour key and histogram *X-axis* individual nematodes accounted for and *Y-axis* the times that the number (account) is repeated

During the 3 years of the study, seven of the eight feeding groups proposed by Yeates [21] were identified; bacterial feeding (BF), plant feeding (PF), fungal feeding (FF), omnivorous (OM), predacious (PR), bacterial feeding or entomopathogenic (BF OR EN) and fungal feeding or entomopathogenic (FF OR EN). In the absence of a weekly chemical disease management treatment, up to 5, 6 and 6 trophic groups were present for each respective potato genotype (cisgenic Desiree, Desiree and Sarpo Mira) across the 3 years. In the presence of a weekly chemical disease management application, trophic group numbers were identified at up to 6, 5 and 6 for cisgenic Desiree, Desiree and Sarpo Mira respectively. The variability across the 3 years of the field study is evident in Fig. 3. In 2013 (Fig. 3a) only five trophic groups (bacterial feeding (BF), plant feeding (PF), omnivorous (OM), fungal feeding (FF) and bacterial feeding or entomopathogens (BF or EN) were identified with BF and PF dominating more than 80% of the total recorded, with PF significantly dominating (P < 0.01) the chemical treated cultivar over the control samples for each cultivar. Six and seven trophic groups were recorded in 2014 and 2015 respectively, with a high population of BF followed by OM in both years.

With the exception of the PF group, which was statistically different between years (P < 0.001) and in regard to year × disease management (P < 0.01), no significant difference was recorded across the remaining groups for either cultivar/disease management/year studied. Across the study, no predator nematodes were identified in either the Sarpo Mira (control), Desiree (chemical) or cisgenic Desiree (chemical) rhizosphere samples. Across the 3 years examined, weak effects were observed for the impact of disease management on PR (P < 0.13) and year (P < 0.07) and the interaction of year × disease management on BF (P < 0.13) and year for OM (P < 0.08) but overall crop genotype had no significant impact on the occurrence of trophic groups observed (P < 0.05).

### Functional guild indices

Examining the degree of colonizer–persister (cp) across the main trophic groups (BF, PF, FF, OM and PR); BF recorded 1–3, FF 2–3, PR recorded 1, 3–5, OM 4 and 5 and PF 2–4. Evaluating the diversity of nematode functional groups and their respective c–p classification, each index recorded a distinct response (Table 3). For EI, the highest mean was associated with chemical treatment (71) versus the absence of chemical fungicides (49), while the inverted trend occurred with BF_2_ (29/51). In both cases the differential values were significant (P < 0.05). The influence of chemical applications led to the highest mean values recorded with the EI and BF_1_ index (EI; 72, 78, 62 and BF_1_; 93, 90, 67) compared to the respective control values (EI; 50, 56, 41 and BF_1_; 64, 89, 66). In contrast, for BF_2_ the highest values were recorded in the absence of chemical management (50, 44 and 59). At a crop genotype level, Sarpo Mira recorded the lowest mean EI value (52) but subsequently the highest BF_2_ mean (48) and CH (17). The cisgenic Desiree genotype returned the lowest SI mean (41) but the highest BF_1_ (89). Examining the influence of year in more detail, four of the five indices (EI, BF_1_, BF_2_ and SI) presented a significant difference (P < 0.001, P < 0.004, P < 0.001 and P < 0.001 respectively) across the 3 years of the study (Table 4). Examining the values in more detail, 2013 recorded the lowest mean values for the EI, BF_1_ and S1 indices (20, 39 and 16 respectively) and highest with the BF_2_ index (80).

**Table 3:** Effect of potato genotype (Desiree, cisgenic Desiree, Sarpo Mira), disease management (control, chemical treatment) and year (2013, 2014, 2105) on nematode trophic diversity indices [enrichment index (EI), bacterial feeding c–p = 2 (BF_2_), bacterial feeding c–p = 1 (BF_1_), chanel index (CI) and the structure index (SI)] studied based on rhizospheric samples taken from Oak Park field site

Index	Potato genotype	Disease management	2015	2014	2013	Mean	Mean/genotype	Mean/disease management
EI	Desiree	Control	67	83	0	50	61	49*
	Desiree	Chemical	83	86	45	72		71
	Cisgenic Desiree	Control	61	92	14	56	67	
	Cisgenic Desiree	Chemical	97	81	56	78		
	Sarpo Mira	Control	33	87	4	41	52	
	Sarpo Mira	Chemical	95	92	0	62		
	Mean		73	87	20***			
BF_2_	Desiree	Control	33	17	100	50	39	51*
	Desiree	Chemical	17	14	55	28		29
	Cisgenic Desiree	Control	39	8	86	44	33	
	Cisgenic Desiree	Chemical	3	19	44	22		
	Sarpo Mira	Control	67	13	96	59	48	
	Sarpo Mira	Chemical	5	8	100	38		
	Mean		27	13	80***			
CH	Desiree	Control	2	7	0	3	5	16
	Desiree	Chemical	3	1	16	7		6
	Cisgenic Desiree	Control	0	0	33	11	11	
	Cisgenic Desiree	Chemical	0	15	16	10		
	Sarpo Mira	Control	0	3	100	34	17	
	Sarpo Mira	Chemical	0	0	0	0		
	Mean		1	5	28			
BF_1_	Desiree	Control	98	93	0	64	78	73
	Desiree	Chemical	97	99	84	93		83
	Cisgenic Desiree	Control	100	100	67	89	89	
	Cisgenic Desiree	Chemical	100	85	84	90		
	Sarpo Mira	Control	100	97	0	66	66	
	Sarpo Mira	Chemical	100	100	0	67		
	Mean		99	95	39***			
SI	Desiree	Control	89	54	17	53	54	47
	Desiree	Chemical	78	52	34	55		52
	Cisgenic Desiree	Control	53	77	0	43	41	
	Cisgenic Desiree	Chemical	44	70	0	38		
	Sarpo Mira	Control	74	64	0	46	55	
	Sarpo Mira	Chemical	82	67	41	63		
	Mean		70	64	16***			

* P < 0.05, ** P < 0.01, *** P < 0.001

**Table 4:** Analysis of variance for the effect of potato ‘genotype’ (Desiree, cisgenic Desiree, Sarpo Mira), disease management (control, chemical treatment) and year (2013, 2014, 2105) on the trophic diversity indices EI, BF_2_, CH, BF_1_ and SI (see Table 3 legend for explanation of abbreviations), in field site in Oak Park (Carlow, Ireland)

Treatment	EI	BF_2_	CH	BF_1_	SI
Genotype	1.06	0.93	0.45	1.2	1.66 (B) (P < 0.24)
Management	6.52 (P < 0.05)	5.94 (P < 0.05)	1	0.75	0.39
Genotype × management	0.002	0.0008	5.94	0.63	0.90
Year	23.10 (P < 0.001)	21.59 (P < 0.001)	2.49 (A) (P < 0.13)	10.17 (P < 0.004)	22.02 (P < 0.001)
t5%	–	–	3.827	–	2.87
t1%	–	–	4.855	–	3.827

A and B = t value compared against the critical value to 5 and 1% in the same column

**Fig. 4:**
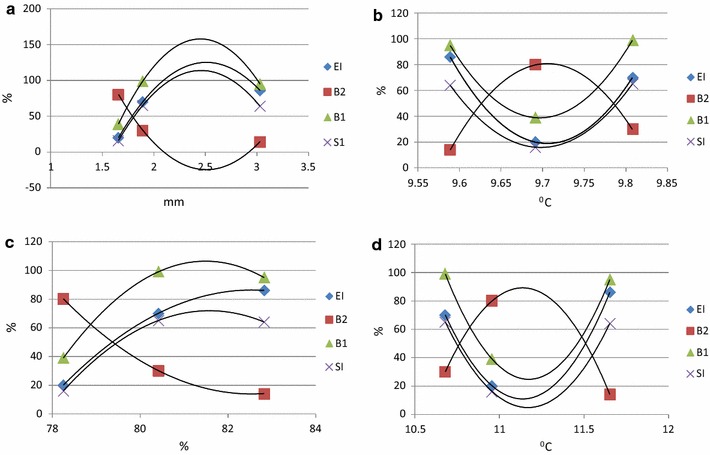
Influence of **a** rainfall, **b** air temperature, **c** relative humidity and **d** soil temperature variables on mean nematode trophic group index values: enrichment index (EI), bacterial feeding c–p = 2 index (BF_2_ = B2), bacterial feeding c–p = 1 index (BF_1_= B1) and structure index (SI) in 2013, 2014 and 2015. Oak Park (Carlow, Ireland)

Investigating potential associations between the mean values obtained for the EI, BF_2_, BF_1_ and SI indices and the recorded weather metrics (air temperature, soil temperature, relative humidity and rainfall) identified consistent polynomial associations for each of the indices studied (Fig. 4), with rainfall (Fig. 4a) and relative humidity (Fig. 4c) impacting similarly on mean index values and correspondingly for the variables of air (Fig. 4b) and soil (Fig. 4d) temperature. For the direct factors of rainfall and soil temperature, inverse associations (BF_2_ vs. EI, BF_1_ and SI) were observed for the indices relative to the factor studied (Fig. 4a, d). This trend was also observed for the indirect factors of relative humidity (Fig. 4c) and air temperature (Fig. 4b).

**Fig. 5:**
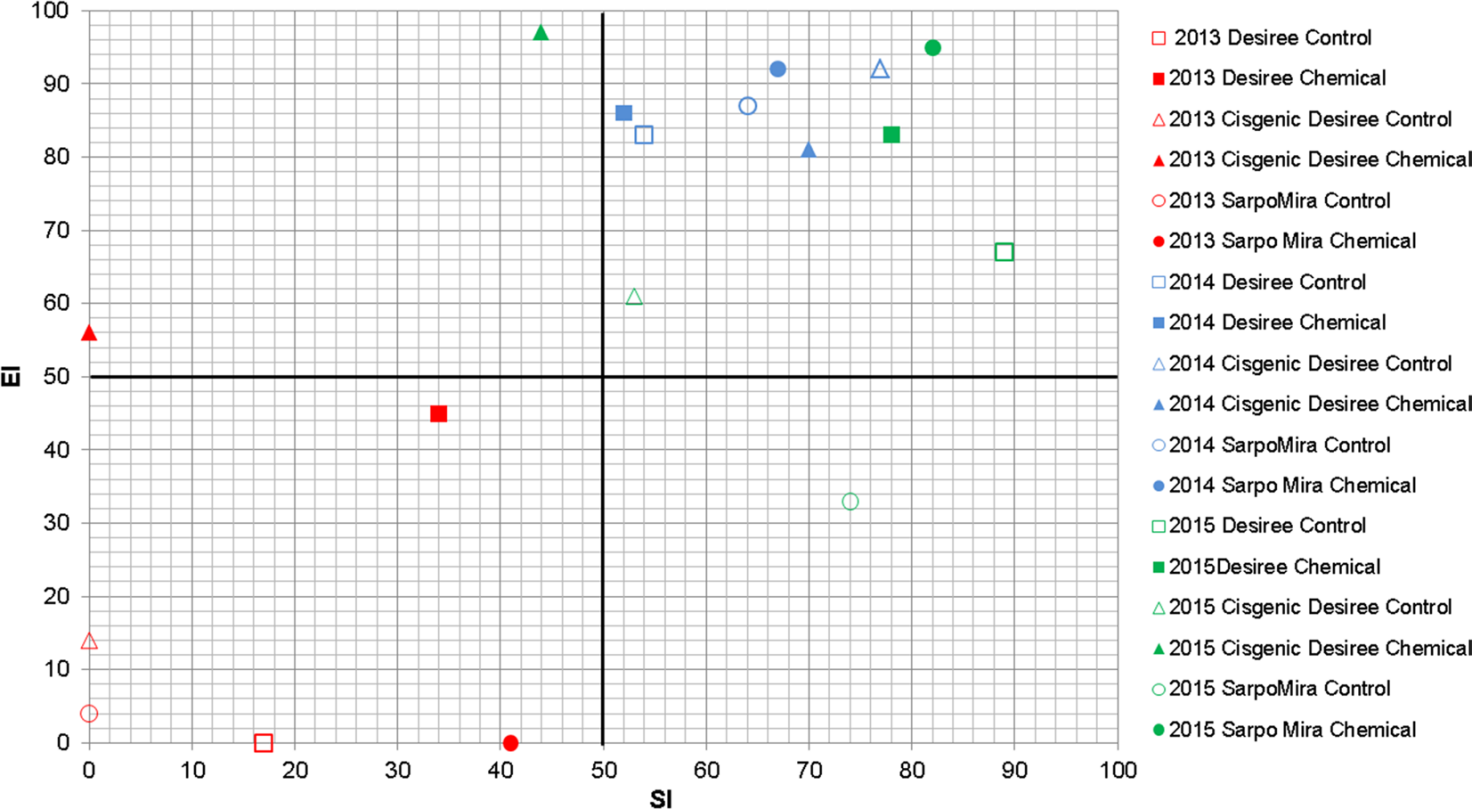
Influence on nematode functional guilds according to the cultivation of potatoes genotypes (Desiree, cisgenic Desiree, Sarpo Mira) treated with different disease management regimes (control, chemical treatment) through the years of 2013, 2014 and 2015. *Up left* (Quadrat A), *up right* (Quadrat B), *down right* (Quadrat C) and *down left* (Quadrat D)/interaction of *EI* enrichment and *SI* structure. Oak Park (Carlow, Ireland)

Examining treatment effects on the basal, structural and enrichment components of the soil food web identified a significant difference (P < 0.001) between EI and SI over time. The construction of nematode profiles for 2013 revealed that food webs for 5 of the 6 treatments (exception being cisgenic Desiree + chemical) positioned within quadrat D (Fig. 5), indicating a depleted and degraded food wed structure. For 2014, all six treatments were plotted to quadrat B, typical of an enrichment condition. In the case of 2015, the final year of the study, all treatments remained in quadrat B, with the exception of the Sarpo Mira control and the cisgenic Desiree + chemical, which positioned in quadrat C and A respectively (Fig. 5).

### Nematode abundance and diversity indices

**Fig. 6:**
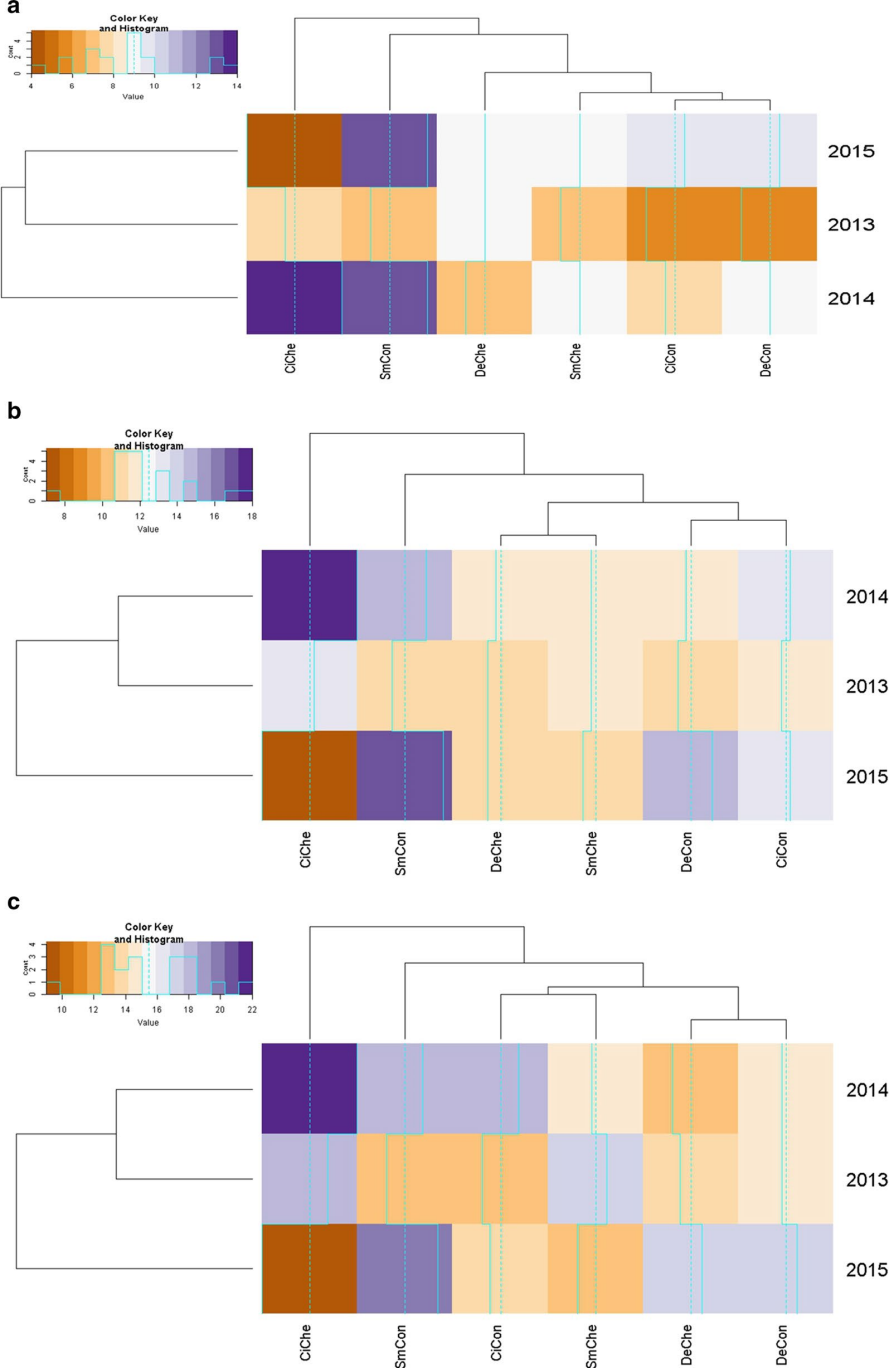
Heatmaps illustrating number of individual nematode **a** family, **b** genus and **c** species identified following extraction from the rhizosphere of potatoes genotypes (Desiree, cisgenic Desiree and Sarpo Mira) cultivated at Oak Park (Carlow, Ireland) under two different disease management regimes (control, chemical treatment) through 2013, 2014 and 2015. Genotype × management interactions are labelled as: Desiree control [DeCon], Desiree chemical [DeChe], cisgenic Desiree control [CiCon], cisgenic Desiree chemical [CiChe], Sarpo Mira control [SmCon], Sarpo Mira chemical [SmChe]. For the colour key and histogram *X-axis* number of individual nematodes accounted for and *Y-axis* the times that the number (account) is repeated

An alternative measure of disturbance considered was the impact of crop genotype and/or disease management treatments on nematode diversity, measured through the abundance of individual nematode family, genus and species and at a species level according to richness (H), evenness (EH) and (D, 1-D and 1/D) dominance indices on a yearly basis through the study. While samples collected from the cisgenic Desiree chemical treatment plots during 2014 and 2013 recorded higher numbers of nematode species and genera than the alternative treatments (Fig. 6), no statistical difference was detected. Taking into account the rare (less frequent-Shannon index) and abundant (dominant-Simpson index) species per sample, the diversity indices returned similar patterns between treatments (Table 5). The mean H index values were >2 for all treatments, irrespective of year, disease management and potato genotype with no significance recorded between treatment; similarly, no significance was returned between treatments in regards to the evenness distribution (EH) of individuals per species present in samples, which was found to be closer to 1 than to 0 for each combination. The uniformity of the mean H and EH values across treatments is illustrated in Additional file 4: Figure S3. In contrast the probability that two nematodes randomly selected from within a sample belonged to the same species (D) was closer to 0 than 1. Lastly, the analyses recorded a statistically similar but high probability (0.84–0.89) of nematode diversity (1-D) across genotypes (per treatment per year) with the number of species (1/D) recorded between 7 and 9 per crop (Table 5).

**Table 5:** Nematode diversity, as per richness (H), evenness (EH) and dominance (D, 1-D, 1/D) indices, arising from samples taken from under different potatoes genotypes (Desiree, cisgenic Desiree, Sarpo Mira) treated with different disease management (control, chemical treatment) through the years of 2013, 2014 and 2015 at Oak Park (Carlow, Ireland)

Index	Potato genotype	Disease management	2015	2014	2013	Mean	Mean/genotype	Mean/disease management
H	Desiree	Control	2.54	2.51	2.46	2.50	2.43	2.43
	Desiree	Chemical	2.54	2.07	2.48	2.36		2.33
	Cisgenic Desiree	Control	2.27	2.62	2.20	2.36	2.30	
	Cisgenic Desiree	Chemical	1.34	2.8	2.58	2.24		
	Sarpo Mira	Control	2.55	2.46	2.26	2.42	2.40	
	Sarpo Mira	Chemical	2.44	2.23	2.47	2.38		
	Mean		2.28	2.45	2.41			
EH	Desiree	Control	0.67	0.66	0.64	0.66	0.64	0.64
	Desiree	Chemical	0.65	0.56	0.64	0.62		0.63
	Cisgenic Desiree	Control	0.63	0.69	0.57	0.63	0.61	
	Cisgenic Desiree	Chemical	0.34	0.75	0.69	0.59		
	Sarpo Mira	Control	0.65	0.63	0.61	0.63	0.65	
	Sarpo Mira	Chemical	0.76	0.58	0.66	0.67		
	Mean		0.62	0.65	0.64			
D	Desiree	Control	0.10	0.10	0.11	0.10	0.11	0.12
	Desiree	Chemical	0.10	0.18	0.09	0.12		0.15
	Cisgenic Desiree	Control	0.15	0.09	0.16	0.13	0.16	
	Cisgenic Desiree	Chemical	0.38	0.08	0.10	0.19		
	Sarpo Mira	Control	0.12	0.13	0.14	0.13	0.13	
	Sarpo Mira	Chemical	0.11	0.15	0.12	0.13		
	Mean		0.16	0.12	0.12			
1-D	Desiree	Control	0.90	0.90	0.89	0.90	0.89	0.88
	Desiree	Chemical	0.90	0.82	0.91	0.88		0.85
	Cisgenic Desiree	Control	0.85	0.91	0.84	0.87	0.84	
	Cisgenic Desiree	Chemical	0.62	0.92	0.90	0.81		
	Sarpo Mira	Control	0.88	0.87	0.86	0.87	0.87	
	Sarpo Mira	Chemical	0.89	0.85	0.88	0.87		
	Mean		0.84	0.88	0.88			
1/D	Desiree	Control	10.41	10.00	9.09	9.83	9.33	8.53
	Desiree	Chemical	9.80	5.56	11.11	8.82		8.38
	Cisgenic Desiree	Control	6.61	11.11	6.25	7.99	8.19	
	Cisgenic Desiree	Chemical	2.67	12.50	10.00	8.39		
	Sarpo Mira	Control	8.45	7.69	7.14	7.76	7.85	
	Sarpo Mira	Chemical	8.80	6.67	8.33	7.93		
	Mean		7.79	8.92	8.65			

### Nematode families and genus as a bio-indicator of environmental disturbance

**Fig. 7:**
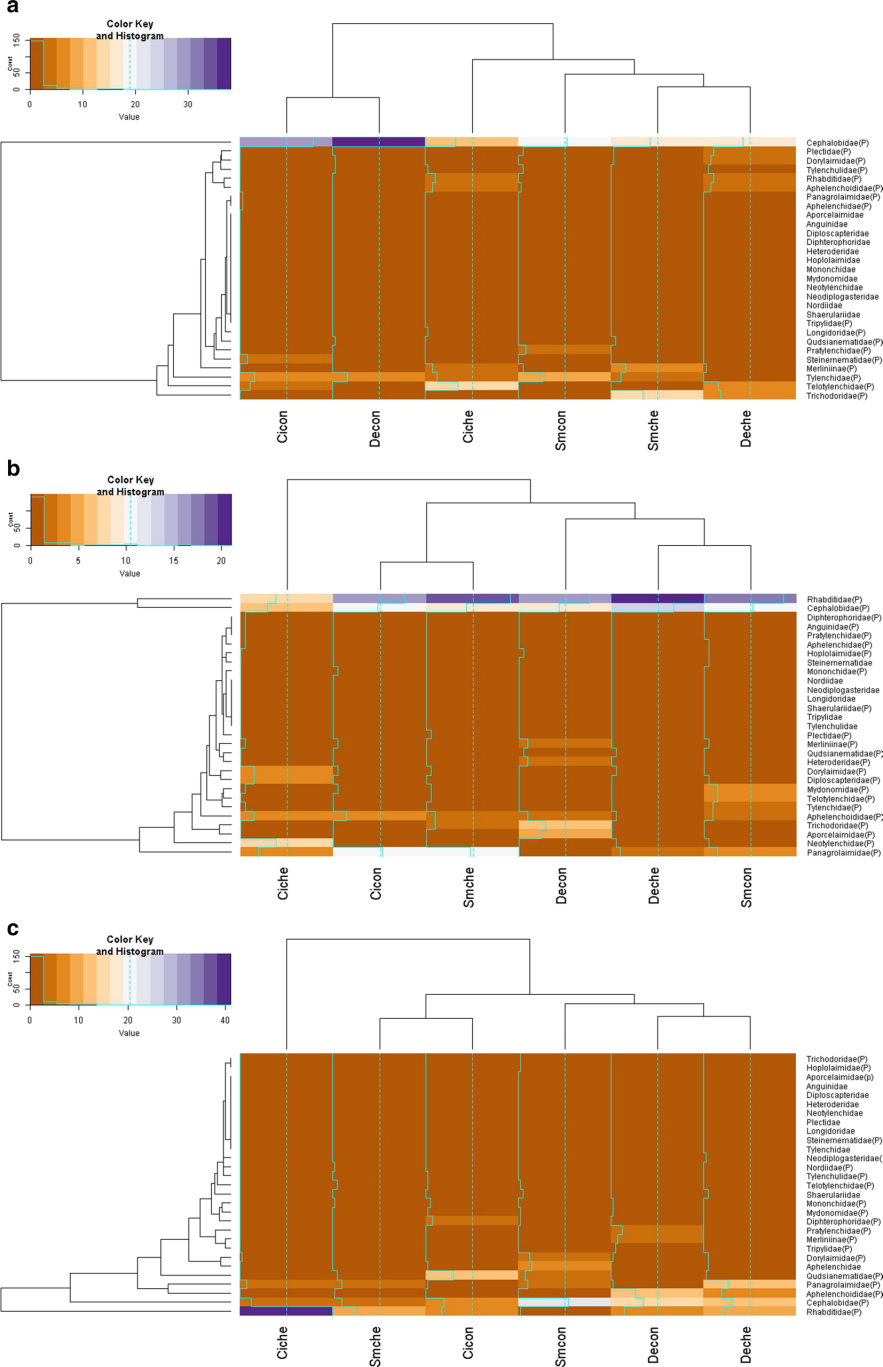
Heatmaps illustrating the distribution of nematode families relative to each potato genotype × management interaction (Desiree control [DeCon], Desiree chemical [DeChe], cisgenic Desiree control [CiCon], cisgenic Desiree chemical [CiChe], Sarpo Mira control [SmCon], Sarpo Mira chemical [SmChe]) for 2013 (**a**), 2014 (**b**) and 2015 (**c**). Oak Park (Carlow, Ireland). For the colour key and histogram *X-axis* number of individual nematodes accounted for and *Y-axis* the times that the number (account) is repeated

Up to 29 distinct families were associated across all treatments evaluated over 2013, (Fig. 7a), 2014 (Fig. 7b) and 2015 (Fig. 7c). There was no significant difference (P < 0.05) between the family, and genus, nematode numbers of cisgenic Desiree [16 (family, control treatment) vs. 17 (family, chemical treatment) and 26 (genus, control treatment) vs. 27 (genus, chemical treatment)] and its comparator Desiree genotype [17 (family, control treatment) vs. 15 (family, chemical treatment) and 27 (genus, control) vs. 25 (genus, chemical)], irrespective of the absence/presence of disease management strategies. For Sarpo Mira, there was a decrease in numbers following chemical treatment [22 (family, control treatment) vs. 14 (family, chemical treatment) and 31 (genus, control treatment) vs. 26 (genus, chemical treatment)]. Sorensen coefficient values calculated for nematode families within each potato genotype (Table 6) indicated substantial overlap between treatments: 0.63 for Desiree control vs. chemical, 0.79 for cisgenic Desiree control vs. chemical and 0.67 for Sarpo Mira control vs. chemical during the 3 years. Factoring the influence of time, coefficient values were calculated for each respective permutation of genotype and disease management (Table 6). Examining equivalence at the family level, Sarpo Mira coefficient values were similar through the 3 years of the study (0.55–0.57) compared to the more variable Desiree (0.50–0.63) and cisgenic Desiree (0.43–0.73). Independent of the regime deployed, for 2013 a cisgenic Desiree vs. Desiree comparison returned a CC = 0.53, in contrast to 0.34 for cisgenic Desiree vs. SarpoMira. For 2014, values ranged from 0.37 to 0.47, while from the final year (2015), cisgenic Desiree and SarpoMira shared 50% of nematode families sampled.

**Table 6:** Sorensen coefficients calculated on comparisons within^a^ potato genotypes (control, chemical treatment) and between^b^ potato genotypes for each individual year (2013, 2014 and 2015) and total during the 3 years for nematode families and genus identified from study completed at Oak Park (Carlow, Ireland)

	Comparison^a^	Year			Total	Comparison^b^	Year			Comparison	Year		
		2015	2014	2013			2015	2014	2013		2015	2014	2013
Family	Desiree control vs. chemical	0.63	0.50	0.53	0.63	Desiree vs. Cisgenic Desiree	0.48	0.37	0.53	Control vs. chemical	0.36	0.54	0.41
	Cisgenic Desiree control vs. chemical	0.43	0.73	0.43	0.79	Desiree vs. SarpoMira	0.49	0.47	0.62				
	Sarpo Mira control vs. chemical	0.55	0.57	0.55	0.67	Cisgenic Desiree vs. Sarpo Mira	0.50	0.36	0.34				
Genus	Desiree control vs. chemical	0.46	0.58	0.18	0.50	Desiree vs. Cisgenic Desiree	0.48	0.36	0.69	Control vs. chemical	0.32	0.48	0.31
	Cisgenic Desiree control vs. chemical	0.40	0.58	0.38	0.63	Desiree vs. SarpoMira	0.47	0.40	0.74				
	Sarpo Mira control vs. chemical	0.28	0.52	0.64	0.64	Cisgenic Desiree vs. Sarpo Mira	0.49	0.41	0.64				
							*0.48*	*0.39*	0.69***				

Italic values indicate Sorensen coefficients for nematode genus identified in 2015, 2014 and 2013 between potato genotypes

* P < 0.05, ** P < 0.01, *** P < 0.001

The relative uniformity in regards to the distribution of nematode families across the treatments with respect to each year is illustrated in Fig. 7. In more detail, on a year-by-year basis 2013 was characterised by seventeen families (Fig. 7a) with the *Cephalobidae* abundant in all treatments evaluated with more *Cephalobidae* individuals noted in the control treatments independent of the crop evaluated (38, 32 and 20 for Desiree control, cisgenic Desiree control and SarpoMira control respectively). Only a nominal number of the *Rhabditidae* family were recorded while seven families associated with plant feeding (*Tylenchulidae, Tylenchidae, Telotylenchidae, Trichodoridae, Merliniinae, Longidoridae, and Pratylenchidae*) were counted. For 2014 (Fig. 7b), 23 families were detected with an abundance of the *Rhabditidae* (8–21 members) family recorded along with the *Cephalobidae* family (6–14) at the same time and members of a third nematode bacterial feeding, the *Panagrolaimidae* (2–11) dominating especially in cisgenic Desiree control and SarpoMira chemical derived samples (Fig. 7b). As with 2013, seven plant feeding families were detected (*Heteroderidae, Hoplolaimidae, Merliniidae, Trichodoridae, Pratylenchidae, Tylenchidae, Telotylenchidae*). The *Heteroderidae and Hoplolaimidae* families were present in Desiree control samples and the *Hoplolaimidae* family was only found associated with the Sarpo Mira control sample. The presence of two FF or EN (*Neotylenchidae* and *Sphaerulariidae*) was also identified. For 2015 (Fig. 7c), 20 families were listed with a similar ratio of members of the family *Rhabditidae* (average 12) and *Cephalobidae* (11) and occurrence of the *Panagrolaimidae* (1–11) family recorded across treatments. However, the distribution for *Rhabditidae* and *Cephalobidae* appeared dependent on the interaction of genotype and treatment (e.g. cisgenic Desiree chemical reached a maximum of 41 members as represented by the blue coloured cells in Fig. 7c). The occurrence of BF or EN nematode family members (*Steinernematidae*) was noted in Desiree chemical and SarpoMira control and FF or EN (*Neotylenchidae*) in Desiree and SarpoMira chemical. Five plant feeding families were recorded (*Merliniidae, Telotylenchidae, Tylenchulidae*, *Hoplolaimidae and Trichodoridae*) in total.

**Fig. 8:**
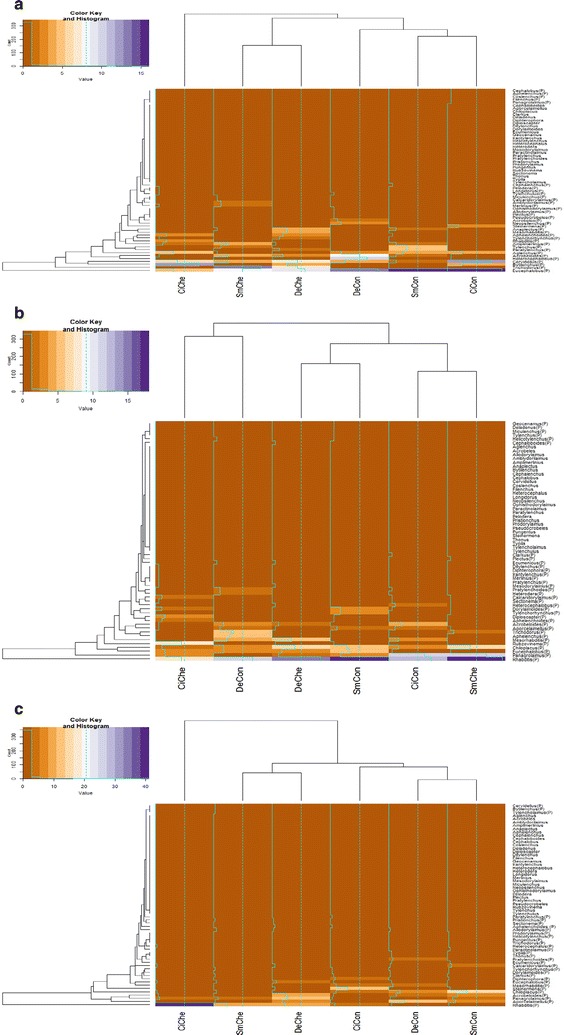
Heatmaps illustrating the distribution of nematode genus relative to each potato genotype × management interaction (Desiree control [DeCon], Desiree chemical [DeChe], cisgenic Desiree control [CiCon], cisgenic Desiree chemical [CiChe], Sarpo Mira control [SmCon], Sarpo Mira chemical [SmChe]) for 2013 (**a**), 2014 (**b**) and 2015 (**c**). Oak Park (Carlow, Ireland). For the colour key and histogram *X-axis* number of individual nematodes accounted for and *Y-axis* the times that the number (account) is repeated

Sixty-two individual genera were identified across the 3 years of the experiment, with 35, 34 and 31 individual genera identified per year, 2013, 2014 and 2015 respectively (Fig. 8a–c). In relation to the Sorensen coefficient within potato genotypes, 50% of genera identified were equivalent between the chemical and control treatment for Desiree and 63 and 64% for the same treatments with cisgenic Desiree and Sarpo Mira respectively (Table 6). As with the nematode family assessment, examining the coefficient values relative to each year of the study identified a broad range from 0.18 to 0.64 when comparing the impact of control vs. chemical treatment across the three potato genotypes studied. In addition, the overlap of genus between potato genotypes (irrespective of chemical treatment) ranged from 0.36 to 0.74, with a clear statistical difference (P < 0.001) between year (Table 6). Temporally, a large overlap (0.64–0.74) of genus was noted in 2013 between potato genotypes; which contrasted with 2014 (0.36–0.41) and 2015 (0.47–0.49). Differences on the presence/absence of specific genera were also evident. For example, *Clarkus* (*Dorylaimia*—2015) and *Pratylenchoides* (*Tylenchida*—2014) were isolated from the Desiree and cisgenic Desiree control plots, with *Clarkus* (*Dorylaimida*—2015) also isolated from SarpoMira—chemical treated plots (Fig. 8b, c).

## Discussion

Conducted over 3 years the goal of this study was to develop an initial baseline on the level of nematode abundance and diversity related to specific potato cropping systems, thereby addressing a knowledge deficit that currently exists in the literature. In particular, it was hypothesised that the cultivation of GM cisgenic Desiree potatoes would not impact significantly on the abundance and/or diversity of non-target soil nematodes. Additional contributory factors that were investigated related to weather variability and the management protocols adopted in regards to the presence/absence of chemical control measures against *P. infestans*, the causative organism of potato late blight disease.

### Succession ecological indices

Nematode ecological succession usually progresses in an orderly and predictable manner unless set back by an environmental disturbance such as cultivation, pollution or nutrient enrichment [22]. The maturity index as originally proposed [38] along with its modifications MIMO, ∑MIMO besides of PPI and ratio PPI/MI have been used previously for monitoring different kinds of disturbance [39, 40], including the cultivation of GM crops [41, 42]. In this 3-year study, and based on the protocol undertaken, the rhizospheric nematode community did not register any significant effect with the cultivation of the cisgenic Desiree line compared to its comparator, cv. Desiree in the presence or absence of fungicide management. Neither was there a significant difference between crop management or the crop cultivars Desiree and Sarpo Mira, which is significant in light of the disparate genetic background of both cultivars and the fact that cv. Sarpo Mira possesses five genetic sources of resistance to *P. infestans* [9].

Although no significant difference was noted in this study in regards to rhizospheric inhabiting nematodes, a similar outcome was reported in regards to the effect of transgenic insect resistant corn on nematode assemblages [41], which was not based on rhizospheric samples. While the results of this study relate to the rhizosphere, it is worth noting that the complexity of the interactions between roots, their exudates and associated soil microorganisms continues to be elucidated [43].

The imprecision of the MI as a quantitative tool has been discussed in previous studies [40, 44] since high MI values, equating to undisturbed conditions, are conditioned by rare K-selected persisters (less disturbed) but with high c–p (3–5) or predominant r-selected opportunistic colonizers with c–p = 1 (enriched) [40]. Therefore, high values in one scenario may mask an accurate estimation of what is actually occurring in regards to nematode diversity. On the other hand, the low MIMO index values obtained in 2013 (in comparison with 2014 and 2015) indicated that the 2013 nematode communities were experiencing an environmental stress, which was irrespective of potato genotype and chemical management applied. It must be acknowledged that the change in land management may have been an influencing factor, with the AMIGA site having previously been a low managed grass pasture for ~10 years, before being used for potato cultivation. However, it is important to note that the rotation strategy adopted in this study ensured that for each year plots were positioned on original grass pasture, thereby ensuring that each year had effectively the same ‘starting point’ in regard to the status of the ground on which the plots were sown. Nematode communities with c–p = 2 have been associated with a limitation of resources, adverse environment conditions or recent contamination [45]. Therefore, while we hypothesise that the index values attained for 2013 were a product of the unfavourable weather conditions, which may have driven the increment in generalist opportunist nematodes (c–p = 2), it is not possible to determine what kind of nematode succession was present, since c–p = 2 are formed by both bacterial (remain of the primary succession) and fungal feeder (secondary succession or primary, depending on the nutrient status C:N ratio) [46]. A qualitative analysis of the maturity indices did indicate differences between the potato genetic background and their interaction with the disease management strategies (the no spray control vs. weekly chemical applications). For example, while for the MI, which encapsulates all free living nematodes, the three genotypes showed a similar tendency, when the members with a c–p = 1 are removed (MIMO) or included the PPI and the PPI/MI ratio, both Desiree and cisgenic Desiree reported comparable tendencies in contrast to Sarpo Mira, which has a different genetic background to that of cv. Desiree. This would indicate that both the Desiree genotype and the cisgenic Desiree genotype studied here interact with and regulate their respective rhizobiomes (likely via root exudates [43] in the same manner and the variability being recorded is inter- as opposed to intra-cultivar specific.

As concluded by Neher [22] the natural ecological succession can be setback by many factors, however, we point out that the level and type of response obtained will depend greatly on environmental conditions (rain and soil temperature as direct factors and environment temperature and relative humidity as indirect factors) in the moment that the experiment is carried out. Soil temperature and moisture have already been identified as primary abiotic factors impacting on nematode distribution and abundance [20]. Here the MI was found to be conditioned by weather conditions and weakly by disease management while it was the MIMO index which was more affected by climatic variation than potato genotype and crop management. Darby et al. [39] showed that the composition of nematode communities (when measured through MI) differ greatly between geographic locations with disparate weather conditions. In this study we detected over the 3 years an influence of weather conditions on community composition, although it is important to clarify that the study was completed at a single geographic location, hence reducing the variability associated with soils from distinct geographic places. Any follow up study should include additional locations in order to comprehensively address this recorded trend.

### Trophic groups

Trophic group absolute value, without distinguishing between c–p, is another method with which to investigate nematode trophic structures [27, 47, 48]. Here no statistical difference was identified in quantitative values between the cisgenic Desiree and either its genetic comparator, cv. Desiree or the alternative genotype Sarpo Mira, plus/minus chemical management practises. Qualitatively, differences were identified. The absence, presence, reduction or increment of trophic groups has been associated with the level of susceptibility or tolerance that some nematode groups experience [47]. Based on the analysis completed in this study, the presence/absence of PR appeared to have been more influenced by the application of chemical fungicides in the disease management regimes and the weather patterns than by the potato genotype. For example, the absence of PR and FF and the increase of BF and PF in all treatments (chemical and control) in 2013 in comparison to 2014 and 2015 could be associated more with limited resources (stress conditions) due to the scarcity of precipitation and the high air and soil temperatures, which occurred through 2013 and would have favoured those nematodes less sensitive to environmental disturbance. In contrast, the weather conditions of 2014 and 2015 were more supportive of an enrichment condition, which can be linked with the reduction of PF (and increase of BF and OM). This would arise from the activation of soil biological processes, hence increasing food resources for the nematode populations [27].

Here the application of fungicides through the chemical management practises, parallels a decrease in the PR group in both Desiree genotypes but it is not possible to associate it directly with a specific active ingredient as different fungicides were applied relative to plant growth stage and the incidence of late blight disease into the site. However, the work correlates with results reported by Smith et al. [49] who examined the impact of the Benomyl™ systemic fungicide on prairie tall grass and it also relates to the use of herbicide [27], which combined, reinforces the theory that the PR nematode group is highly sensitive to chemical disturbance. The only contradiction to this is the fact that the same response was not recorded with Sarpo Mira, suggesting that this cultivar can possibly counteract the negative impact on the PR group; possibly due to the extreme growth vigour of the cultivar, thereby presenting a larger biomass for the PR group to prey on [50]. In this study, cv. Sarpo Mira recorded a higher PPI and PPI/MI ratio under disease management strategies relative to Desiree and cisgenic Desiree. Of interest, Bonger and Ferris [31] determined that the occurrence and abundance of PPI is largely determined by the community structure, host status and critically the vigour of plants growing in the soil. This phenomenon is supported here where the presence of the PR group could be influenced by the reduction/elimination of the prey (PPI) taking into consideration plant vigour, the chemical applications or the interaction of both. In this study, the PPI index as previously discussed was influenced somewhat by potato genotype and disease management (but not weather conditions); the separation in trophic groups here suggests that PF is most likely influenced by all three factors. It is also worth considering that as the vigour of cv. Sarpo Mira induced a different nematode community structure, the inclusion of such a vigorous phenotype in an integrated pest management plan may generate a balanced community (PR and PPI) and hence induce consistent levels of suppression against distinct pathogenic nematodes. While there was no evidence of pathogenic nematodes in the field used in this study, bearing in mind a recent review [51] on the role of predacious nematodes in the biological control of plant parasitic nematodes, this phenomenon requires further study.

Indifferent to maturity indices, where basically the nematode community is separated into two groupings, colonizers and persisters, the separation of the nematode community using trophic groups provides a valuable insight into the complexities of the rhizosphere. As such, we hypothesise, based on this preliminary study that trophic groups such as PR can be influenced by disease management strategies by weather conditions and possible by the plant vigour and that FF or EN, BF or EN and OM are more influenced by weather conditions with BF affected by the interaction of weather and disease management.

### Functional guild

The combined analysis of the response of the nematode community through its feeding type or trophic group [21] and its life history strategy [38], measured as its functional guild [46] is another way of evaluating the response of the nematode community to environmental disturbance factors. Comparing the disease management regimes independently of potato genotype showed a significant difference existed for the EI and BF_2_ indices in regard to presence/absence of fungicide applications. As shown previously with the trophic groups, fungicide applications also altered the structure of the soil food web. In this study, the weekly chemical fungicide treatments generated an enrichment condition given per an increment of BF_1_ and reduction in CH. In contrast, the corresponding control treatment highlighted a more basal condition which included recovery from a moderate disturbance, through tillage and fertilizer operations as part of the standard management of the site.

Predation and competition among trophic levels provide “top–down” regulation of food web structure and function [45]. The significant differences noted here between years for the EI, BF_1_, BF_2_ and SI and the different treatment on the distinct quadrants would come to confirm not only the significance of year-to-year disturbances as per the Succession Ecological Indices and the Trophic Groups but also the type of disturbance. The information obtained through the assessments of the functional guilds support the conclusion of Cesarz et al. [52], stating that knowledge on functional guilds proves a better understanding about soil alterations.

### Nematode taxonomy, abundance and diversity indices

The nematode community structure was examined in both a qualitative and quantitative manner. Based on the results from this study the association between the *Clarkus* genus (Family *Mononchidae*) and the entomopathogenic *Steinernema* (Family *Rhabditidae*), *Rubzovinema* (Family *Neotylenchidae*) and *Deladenus* (Family *Shaerulariidae*) genus may serve as a bio-indicator of environmental disturbance through for example the application of chemical fungicide. While the *Trichodorus* (*Trichodoridae*) appear to be relatively tolerant to adverse weather conditions (dry weather) [53] and fungicide application, on the other hand plant parasitic nematodes occur in three widely separated orders: Triplonchida, Dorylaimida and Tylenchida. All triplonchid and dorylaimid plant parasitic nematodes are migratory ectoparasites of roots. Within the Tylenchida however, several different types of plant parasitism can be recognised [54]. In the first year (2013) of this study, migratory ectoparasites (1d) [21] of roots (*Longidoridae*, *Trichodoridae and Tylenchidae*) were identified dominating in samples derived from chemical treated plots. This contrasted with epidermal cell and root hair feeders (1e) [21] and algae lichen feeders (1f) [21] (*Tylenchidae*) as found in the non-chemical treated samples. As 2013 was characterized by unfavourable climatic conditions for nematode community structure as associated by an abundance of members of the *Cephalobidae* family, 2014 and 2015 were years where the *Rhabditidae* family dominated along with a distribution of Tylenchidas with distinct types of plant (1a–f) [21] and *Trichodorus* ectoparasites. The identification of the *Aporcelaimellus* genus during the years of higher rainfall (2014 and 2015) along with a general increase in the numbers of omnivorous nematodes, would support previous hypothesis by Porazinska et al. [55], whereby the correlation of soil moisture with the presence of omnivorous nematodes is more long term than temporary.

As distinct nematode family, genus or species respond in different ways to disturbed soil management practises [40, 56] or environmental perturbations [26, 46, 53], taxonomic analysis focussed at a genus level can be considered fundamental [40, 57], in regards to quantifying the impact of crop genotype cultivation on rhizospheric nematode diversity. From observations made in this study, such comparative taxonomic analysis to the level of genus were most sensitive in detecting temporal differences across the 3 years of the study, with the adverse conditions of 2013 appearing to induce a similar population of genus across the treatments.

In our study, sequencing a fragment of the 18 SSU rDNA gene sufficiently discriminated between nematode populations across the different disease management treatments and weather conditions. Indeed, the number of genera detected in this study exceed that recorded in previous GM-related studies that relied solely on morphological identification [42, 58]. Similarly, alternative DNA-based detection techniques (e.g. T-RFLP) have also proven versatile at capturing more information than classical morphological analysis [59]. Sample pooling to facilitate sequencing has been recently demonstrated in regards to the high throughput sequencing of soil nematode communities [60]. For the work presented here, the approach of sequencing clones from a unique composite sample did provide a detailed representation of nematode diversity as supported by the richness, high value of equitability (EH) where the distribution of species inside the samples was more than 60%. This point is supported by the other parameter affecting nematode diversity, Simpson diversity index (1-D), in which a probability up to 89% was obtained, indicating the high probability that two individuals randomly selected from the same sample belong to different species. Further support is provided by the completed rarefaction analysis, which indicated the high levels of nematode species and genus richness obtained relative to the sampling process adopted. In light of the recent advancements in next generation sequencing (NGS) technologies, and the ever-reducing costs of applying these processes, future studies that use NGS will provide complementary insight into the observations made here in developing a robust database while also elucidating further the levels of nematode diversity within the potato rhizosphere.

Overall, this study has generated a baseline dataset accounting for nematode abundance and diversity for GM potato cultivation practices over 3 years. Capitalising on this resource, evaluations concluded that year of analysis exerted the largest impact on nematode diversity and that the cultivation of a cisgenic *P. infestans* resistant potato genotype had no significant effect on nematode diversity and community structure that was any greater than its comparator potato genotype cv. Desiree. Separately, the knowledge base generated here, provides an opportunity to develop specific bio-indicators to assist future environmental studies, specifically in regards to the cultivation of conventional/genetically engineered potatoes and/or fungicide applications. Taking into consideration that the response of a bio-indicator is dependent upon the interaction of multiple factors (e.g. host genotype × phenotype, weather conditions, crop management practises, presence/absence of crop pathogens), to build upon the outputs of this study, first steps should consider validating the output from this study across multiple locations for specific nematode families/genus as indicated here. From that, a paradigm should be established with multiple factors included, relative to the variables of the studies being used for data input and which are known to affect the environment, which is relevant to the bio-indicator. From here, the robustness of the emerging model can then be tested across an expanded trial system prior to its implementation as a diagnostic bio-indicator for environmental studies.

## Conclusions

- Cultivation of the cisgenic Desiree line studied here had no significant effect on nematode community diversity and/or structure relative to that recorded for its comparator, cv. Desiree. Differences that were recorded were inter-genotype specific.
- Fungicide applications can influence nematode community structures and this can be exasperated by extreme weather conditions. However, it would appear that this effect can be countered by the vigour of the plant being treated.
- The Maturity indices are merely an indicator of a disturbed environment and require the inclusion of functional guild and taxonomic data to accurately quantify levels of disturbance in potato ecosystems.

## Additional files

**Additional file 1: Figure S1.** Flowchart detailing the field design for the AMIGA study across 2013, 2014 and 2015, potato genotypes included and corresponding treatments (* IPM was additional strategy in the AMIGA project but which was not included as a treatment in this study). Post-harvest molecular analysis is also detailed.

**Additional file 2: Table S1.** Summary of indices (Ecological succession, Functional guild and Diversity), graphic representation of food web structure and similarity coefficients employed to characterize the nematode community in the rhizosphere of three distinct potatoes genotypes (cv. Desire, cv. Sarpo Mira and cisgenic Desiree) submitted to two different disease management (control, chemical treatment), during field studies across 2013, 2014 and 2015 park, Ireland.

**Additional file 3: Figure S2.** (a) Mean daily rainfall values taken for Oak Park (Carlow, Ireland) during 2013, 2014 and 2015. (b) Mean daily temperature values taken for Oak Park (Carlow, Ireland) during 2013, 2014 and 2015. (c) Mean daily soil temperature values (at 30 cm depth) taken for Oak Park (Carlow, Ireland) during 2013, 2014 and 2015. (d) Mean daily relative humidity values for Oak Park (Carlow, Ireland) during 2013, 2014 and 2015.

**Additional file 4: Figure S3.** Uniformity of values across the Shannon Diversity (H) and Shannon Equitability (EH) indices for samples taken from under different potatoes genotypes (Desiree, cisgenic Desiree, Sarpo Mira) treated with different disease management regimes (control, chemical treatment) through the years of 2013, 2014 and 2015 at Oak Park (Carlow, Ireland).
