# Supplementary material for: A temporal assessment of nematode community structure and diversity in the rhizosphere of cisgenic Phytophthora infestans-resistant potatoes
Source: BMC Ecol. 2016 Dec 1;16:55. doi: 10.1186/s12898-016-0109-5 (PMC5134073; doi:10.1186/s12898-016-0109-5)
Supplement: Supplementary file 1 — Additional file 1: Figure S1. Flowchart detailing the field design for the AMIGA study across 2013, 2014 and 2015, potato genotypes included and corresponding treatments (* IPM was additional strategy in the AMIGA project but which was not included as a treatment in this study). Post-harvest molecular analysis is also detailed. [file 12898_2016_109_MOESM1_ESM.pptx]

## Slide 1
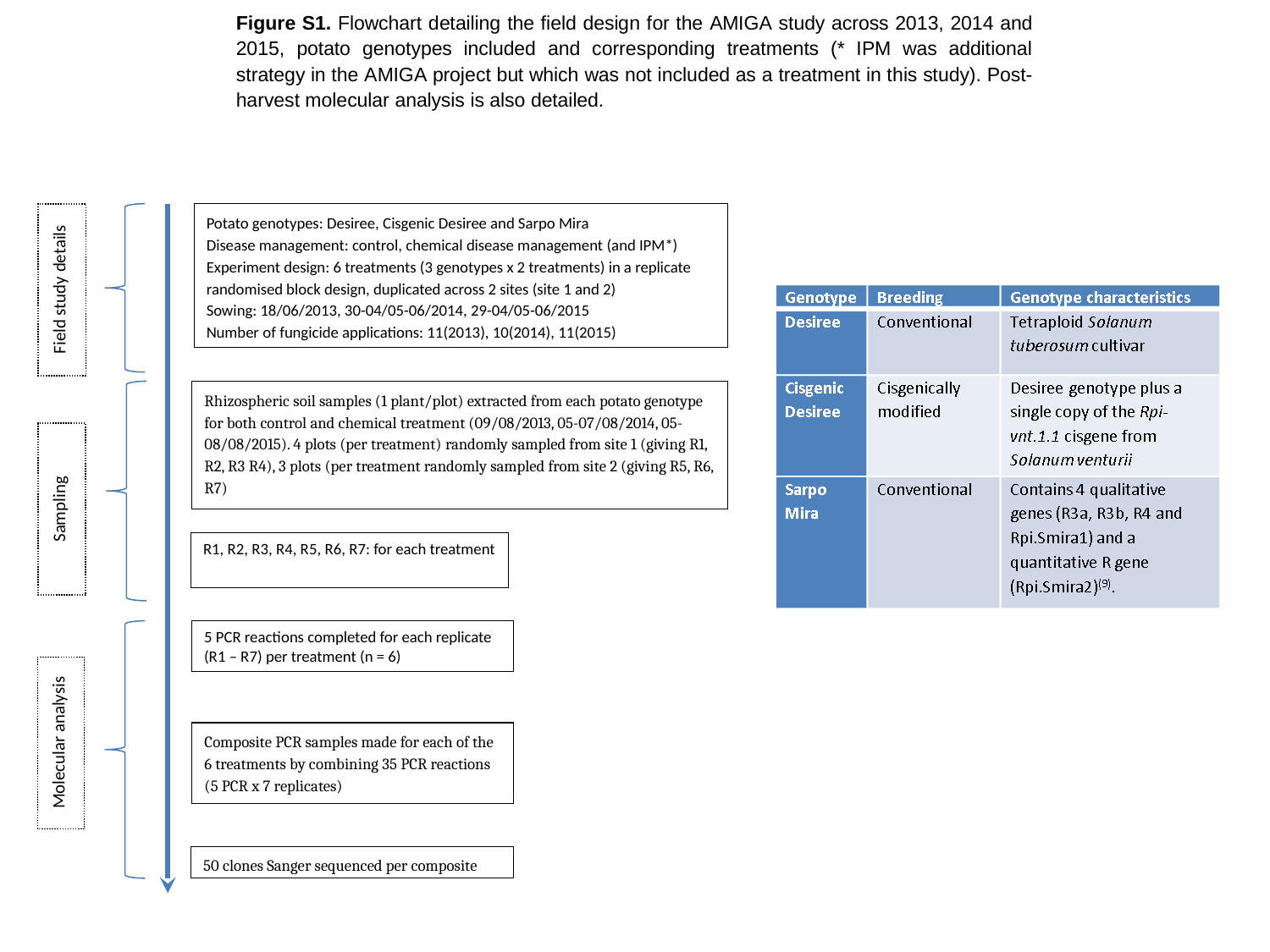

Potato genotypes: Desiree, Cisgenic Desiree and Sarpo Mira
Disease management: control, chemical disease management (and IPM*)
Experiment design: 6 treatments (3 genotypes x 2 treatments) in a replicate randomised block design, duplicated across 2 sites (site 1 and 2)
Sowing: 18/06/2013, 30-04/05-06/2014, 29-04/05-06/2015
Number of fungicide applications: 11(2013), 10(2014), 11(2015)
Field study details
Rhizospheric soil samples (1 plant/plot) extracted from each potato genotype for both control and chemical treatment (09/08/2013, 05-07/08/2014, 05-08/08/2015). 4 plots (per treatment) randomly sampled from site 1 (giving R1, R2, R3 R4), 3 plots (per treatment randomly sampled from site 2 (giving R5, R6, R7)
Sampling
R1, R2, R3, R4, R5, R6, R7: for each treatment
5 PCR reactions completed for each replicate (R1 – R7) per treatment (n = 6)
Molecular analysis
Composite PCR samples made for each of the 6 treatments by combining 35 PCR reactions (5 PCR x 7 replicates)
50 clones Sanger sequenced per composite
