# Supplementary material for: A temporal assessment of nematode community structure and diversity in the rhizosphere of cisgenic Phytophthora infestans-resistant potatoes
Source: BMC Ecol. 2016 Dec 1;16:55. doi: 10.1186/s12898-016-0109-5 (PMC5134073; doi:10.1186/s12898-016-0109-5)
Supplement: Supplementary file 2 — Additional file 2: Table S1. Summary of indices (Ecological succession, Functional guild and Diversity), graphic representation of food web structure and similarity coefficients employed to characterize the nematode community in the rhizosphere of three distinct potatoes genotypes (cv. Desire, cv. Sarpo Mira and cisgenic Desiree) submitted to two different disease management (control, chemical treatment), during field studies across 2013, 2014 and 2015 park, Ireland. [file 12898_2016_109_MOESM2_ESM.docx]

Table S1. Summary of indices (Ecological succession, Functional guild and Diversity), graphic representation of food web structure and similarity coefficients employed to characterize the nematode community in the rhizosphere of three distinct potatoes genotypes (cv. Desire, cv. Sarpo Mira and cisgenic Desiree) submitted to two different disease management (control, chemical treatment), during field studies across 2013, 2014 and 2015 park, Ireland.

| **Index** | **Formula** | **Range** | **Mean** |
| --- | --- | --- | --- |
| **MI [**[**1**](#_ENREF_1)**]** | MI = ∑vi x fi / n, where vi = the colonizer-persister c-p value (1-5), fi = frequency of family i in the sample and n = total number of individuals in the sample | 0-4 | Maturity Index used for assessing the response of nematode assemblages (RNA) to disturbance induced by enrichment and stress conditions. Smaller values being indicative of a more disturbed environment (enrichment and stress) and larger values characteristic of a less disturbed environment |
| **MIMO** | MI modification (removing of the bacterial feeding c-p=1) indicative of enrichment condition. . | 0-4 | Modified Maturity Index used to measure the RNA to disturbance induced by stress effects. Smaller values being indicative of a more disturbed environment and larger values characteristic of a less disturbed environment |
| **∑MIMO** | MI modification (removing of the c-p=1 but inclusion of the PPI) | 0-4 | Modified Maturity Index used to measure the RNA to disturbance induced by stress or nutrient (e.g. nitrogen) immobilisation, as generated by the presence of PPI. Smaller values being indicative of a more disturbed environment and larger values characteristic of a less disturbed environment |
| **PPI [**[**2**](#_ENREF_2)**]** | PPI = ∑ vi x fi / n where vi = is the colonizer-persister c-p value (2-5), fi = frequency of family i in the sample and n = total number of individuals in a sample | 0-4 | Plant Parasitic index is an indicator of the level of nematode feeding on higher plants. Higher values mean abundance of nematode feeding plant. |
| **PPI/MI ratio [**[**2**](#_ENREF_2)**]** | PPI = ∑ vi x fi / n)/ ∑vi x fi / n) | 0-4 | The PPI/MI ratio measures the quantitative relationship between the number of free-living nematodes and plant parasitic nematodes. Ratio values greater than 1.6 means a disrupted level of nutrient uptake by plants (at least temporary) generated by the abundance of PPI in comparison with the free-living nematodes. A value of 0.9 is considered as ideal for optimal nutrient uptake by plants. |
| **EI [**[**3**](#_ENREF_3)**,** [**4**](#_ENREF_4)**]** | 100x (e/e+b) where e=BF_1_ x 3.2 + FF_2_ x 0.8 and b= (BF_2_ + FF_2_) x 0.8  e=enrichment condition  b=basal condition | 0-100% | The Enrichment Index measures the proportion of bacterial feeding (cp=1) and fungal feeding (cp=2) (enrichment conditions) nematodes present in relation to a basal (b) and enrichment (e) condition. Higher values mean an enrichment condition (BF cp=1 and FF=2) and, lower values are indicative of a more basal state (BF cp=2) and (FF cp=2). |
| **SI [**[**3**](#_ENREF_3)**,** [**4**](#_ENREF_4)**]** | 100x (s/s+b) where s= (BF_3_ + FF_3_ + PR_3_) x 1.8 + (BF_4_ + FF_4_ + PR_4_ +OM_4_) x 3.2 + (PR_5_ +OM_5_) x 5  s=structural condition | 0-100% | The Structure Index measures the proportion of free-living nematodes (colonizer-persister) cp=3 to 5 (structural condition) in a nematode community formed by structural and basal conditions. Higher values mean a more structural nematode community while lower values are more associated with a basal condition. |
| **CH [**[**3**](#_ENREF_3)**]** | 100(0.8*FF_2_/BF_1_*3.2 + FF_2_*0.8^)^ | 0-100% | The Chanel Index measures the proportion of fungal feeding nematodes (cp=2) within the nematode community (basal condition) formed by bacterial and fungal feeding (cp=2). Higher values mean an abundance of fungal feeding nematodes in comparison to bacterial feeding nematodes |
| **BF cp=1** | BF_1_=100-CH | 0-100% | In an enrichment condition (BF cp=1 and FF cp=2), the value determines the percentage of bacterial feeding nematodes (cp-1) in comparison to fungal feeding nematodes (cp=2) belonging to a primary or secondary succession or depending on the nutrient status, C:N ratio. |
| **BF cp=2** | BF_2_=100-EI | 0-100% | In an enrichment and basal condition, BF determines the percentage of BF cp=2 property of a basal condition or that which is induced by a stress condition. |
| **Shannon Diversity Index [**[**5**](#_ENREF_5)**,** [**6**](#_ENREF_6)**]** | H = ∑(pilnpi) where pi is the relative abundance of taxon i, | 0-4 | The Shannon Diversity Index (H) is a richness index (number of species present) taking into account rare and abundant species. The utility of this index on population diversity is associated with Shannon Equitability (EH, see below) values with values closer to 4 meaning that many species are present in the sample (singletons, doubletons or abundance though not necessarily more diverse) and lower values less species. |
| **Shannon Equitability [**[**6**](#_ENREF_6)**]** | EH = H / Hmax where Hmax is log_e_S (S = numbers of taxa identified) | 0-1 (or multiply per 100) percentage. | The Shannon Equitability index is an evenness index which determines the distribution of individual species present in a sample. It is complementary to the Shannon Diversity richness index. Values closer to 1 mean that individuals are more homogenously distributed in the sample, hence more diversity. |
| **Simpson Dominance Index [**[**6**](#_ENREF_6)**,** [**7**](#_ENREF_7)**]** | D=∑pi^2^ | 0-1(or multiply per 100) percentage. | The Simpson Dominance Index relates to the number of individuals per species, which is more affected by common or abundant species. The values obtained indicate the probability that two individuals randomized selected from a sample belong to the same species. The closer the value is to 1 the less diversity within the samples and hence more species dominance, while the closer to 0 the index is the more diversity within the sample. This contrasts with the Simpson Diversity index below. |
| **Simpson Diversity Index [**[**6**](#_ENREF_6)**,** [**7**](#_ENREF_7)**]** | Probability of diversity (1-D) | 0-1(or multiply per 100) percentage. | The Simpson Diversity Index is a contrast to the before mentioned Simpson Dominance Index (D). The values calculate the probability that two individuals randomly selected from a sample belong to different species. The closer the value is to 1 the more diversity present in the sample. |
| **Simpson Reciprocal Index [6, 7]** | (1/D) | 0-100 | The Simpson Reciprocal Index calculates the number of species expected depending on the dominance of species in the sample. Higher values imply less dominance within the sample by individual species therefore more diversity is present, while lower values mean more individual species dominance and hence less diversity. |
| **Faunal Analysis [**[**3**](#_ENREF_3)**,** [**8**](#_ENREF_8)**]** | This analysis is based on the feeding habit (trophic group) and life history expressed by colonizer-persister (cp) scale/functional guild. |  | This is the graphical representation of the food web structure, through the interaction of the structure (SI) and enrichment (EI) index values |
| **Sorensen Coefficient [**[**9**](#_ENREF_9)**]** | (CC=100* 2C/A+B where A = numbers of species in the community 1, B= numbers of species in community, C=species in common between both communities) | 0-1(or multiply per 100) percentage. | The Sorensen Coefficient is a tool with which to measure the degree of similarity between communities. The closer the value is to 1 more the communities have in common. |

1. Bongers T: **The maturity index: an ecological measure of environmental disturbance based on nematode species composition.** *Oecologia* 1990, **83**:14-19.

2. Bongers T, Meulen H, Korthals G: **Inverse relationship between the nematode maturity index and plant parasite index under enriched nutrients conditions**. *Applied Soil Ecology* 1997, **6**:195-199.

3. Ferris H, Borgers T, de Goude RM: **A framework for soil food web diagnostics: extension of the nematode faunal analysis concept.** *Applied Soil Ecology* 2001, **18**:13-29.

4. Berkelmans R, Ferris H, M T, A.H.C. VB: **Effects of long-term crop management on nematode trophic levels other than plant feeders disappear after 1 year of disruptive soil management.** *Applied soil ecology* 2003:223-235.

5. Shannon CE, Warren W: **A Mathematical Model of Communication.** . Urbana, IL: University of Illinois Press; 1949.

6. Yeates GW, Bongers T: **Nematode diversity in agroecosystems.** *Agriculture Ecosystems and Environment* 1999, **74**:113-135.

7. Simpson EH: **Measurement of diversity.** . *Nature* 1949, **163**:688.

8. Bongers T, Bongers M: **Functional diversity of nematodes**. *Applied Soil Ecology* 1998, **10**:239-251.

9. Wold H: **Similarity index, Sample size and Diversity.** . *Oecology* 1981, **50**:296-302.
