# Supplementary material for: A temporal assessment of nematode community structure and diversity in the rhizosphere of cisgenic Phytophthora infestans-resistant potatoes
Source: BMC Ecol. 2016 Dec 1;16:55. doi: 10.1186/s12898-016-0109-5 (PMC5134073; doi:10.1186/s12898-016-0109-5)
Supplement: Supplementary file 3 — Additional file 3: Figure S2. (a) Mean daily rainfall values taken for Oak Park (Carlow, Ireland) during 2013, 2014 and 2015. (b) Mean daily temperature values taken for Oak Park (Carlow, Ireland) during 2013, 2014 and 2015. (c) Mean daily soil temperature values (at 30 cm depth) taken for Oak Park (Carlow, Ireland) during 2013, 2014 and 2015. (d) Mean daily relative humidity values for Oak Park (Carlow, Ireland) during 2013, 2014 and 2015. [file 12898_2016_109_MOESM3_ESM.pptx]

## Slide 1
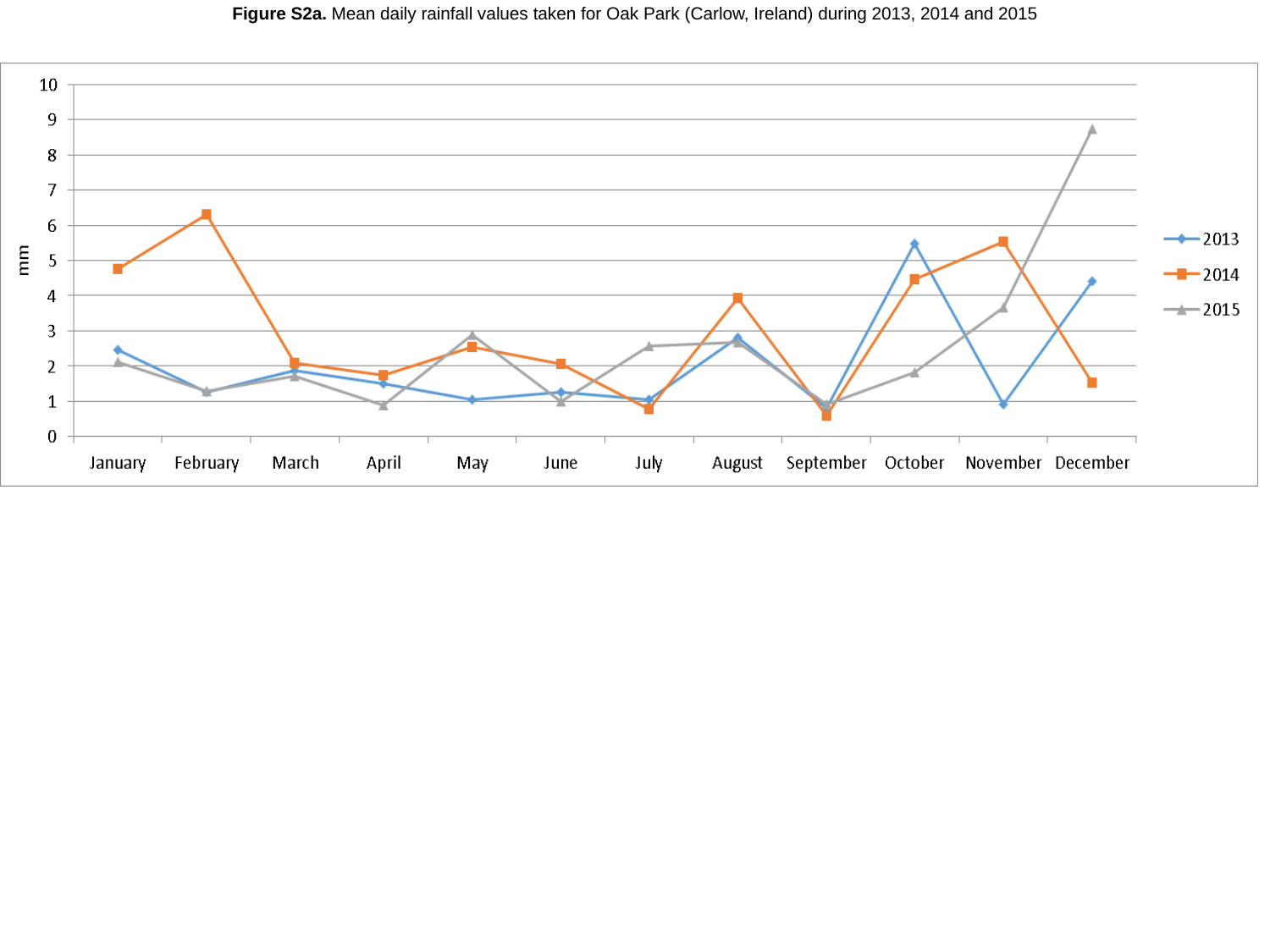

Figure S2a. Mean daily rainfall values taken for Oak Park (Carlow, Ireland) during 2013, 2014 and 2015

## Slide 2
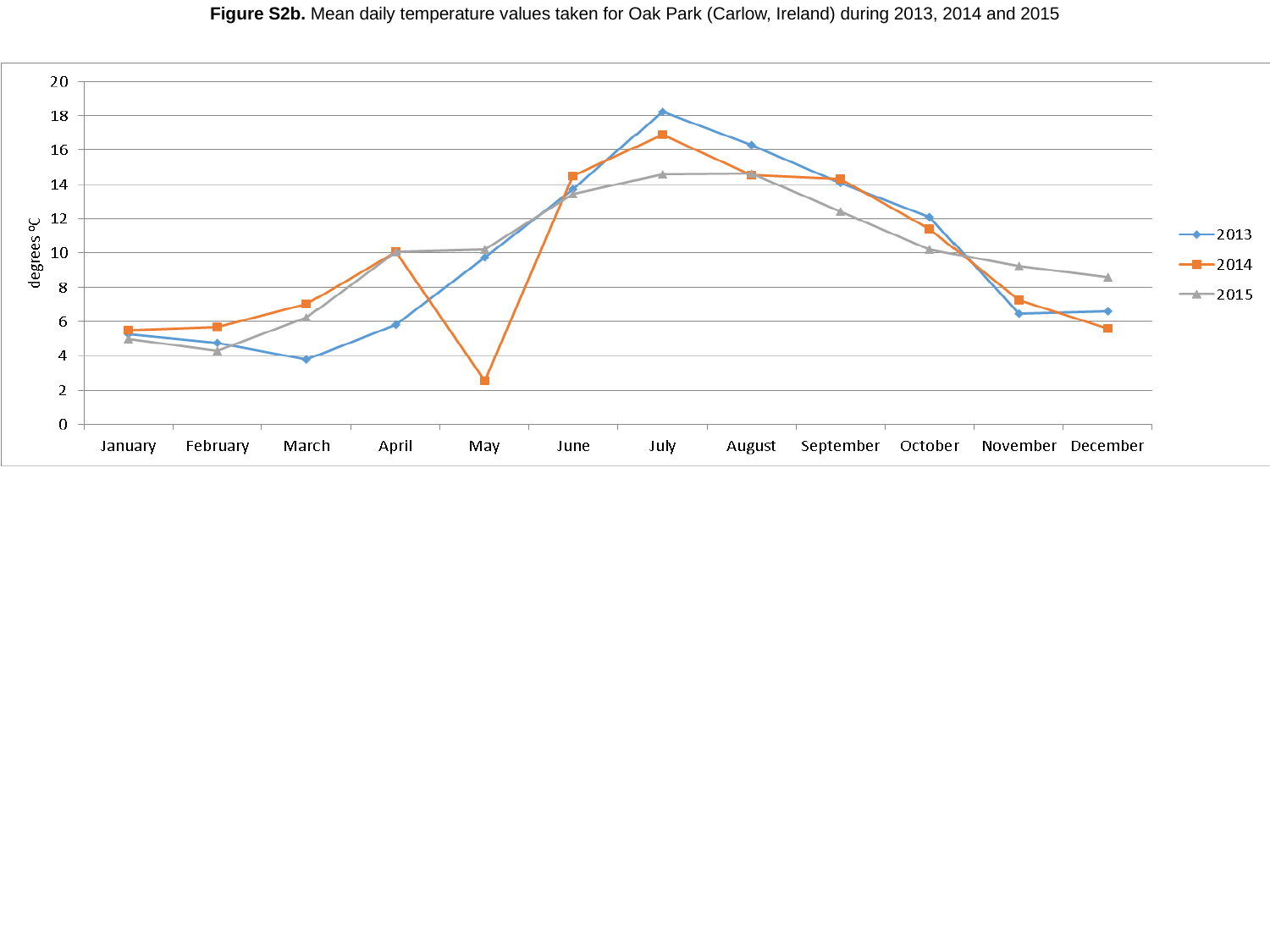

Figure S2b. Mean daily temperature values taken for Oak Park (Carlow, Ireland) during 2013, 2014 and 2015

## Slide 3
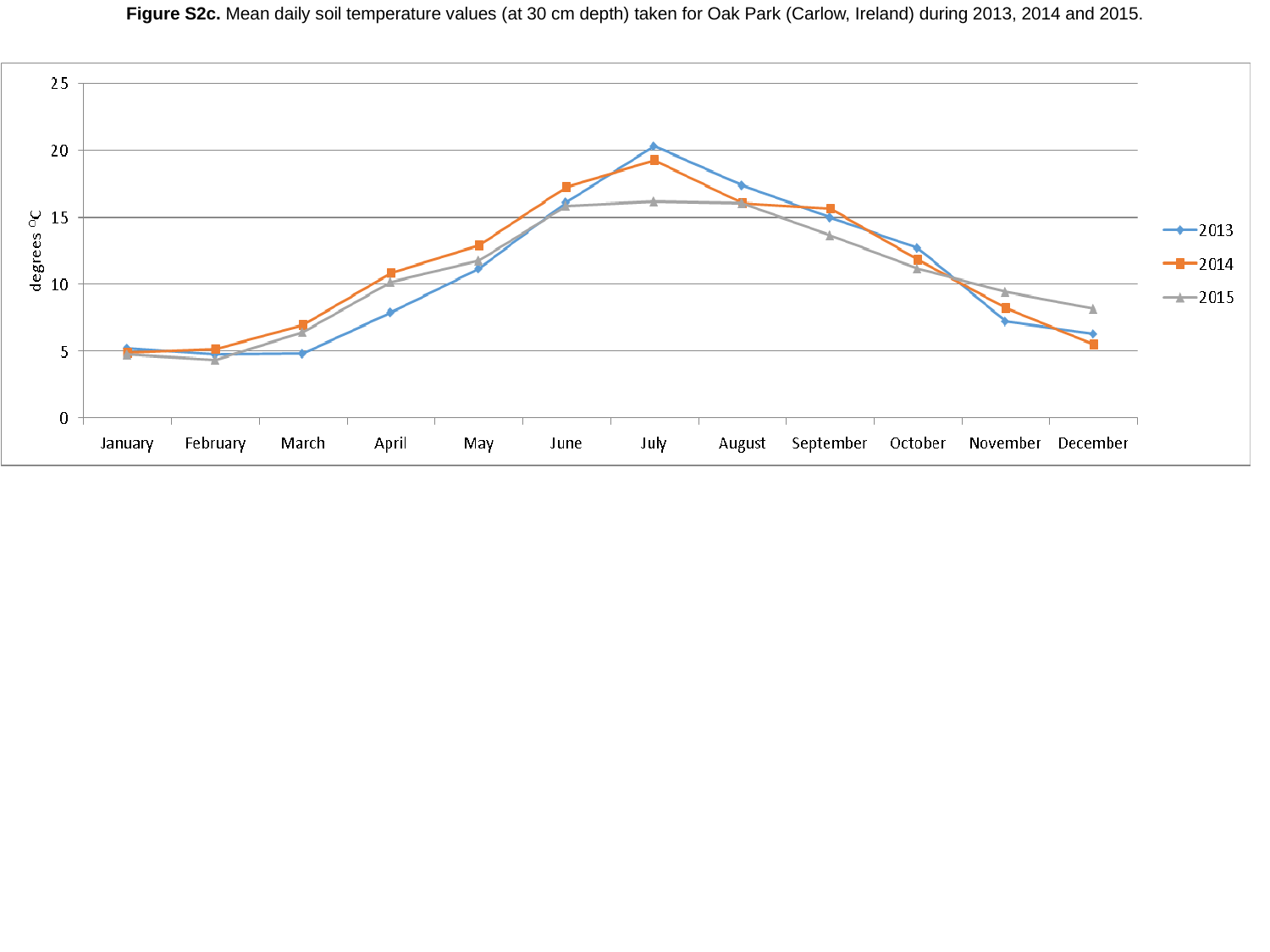

Figure S2c. Mean daily soil temperature values (at 30 cm depth) taken for Oak Park (Carlow, Ireland) during 2013, 2014 and 2015.

## Slide 4
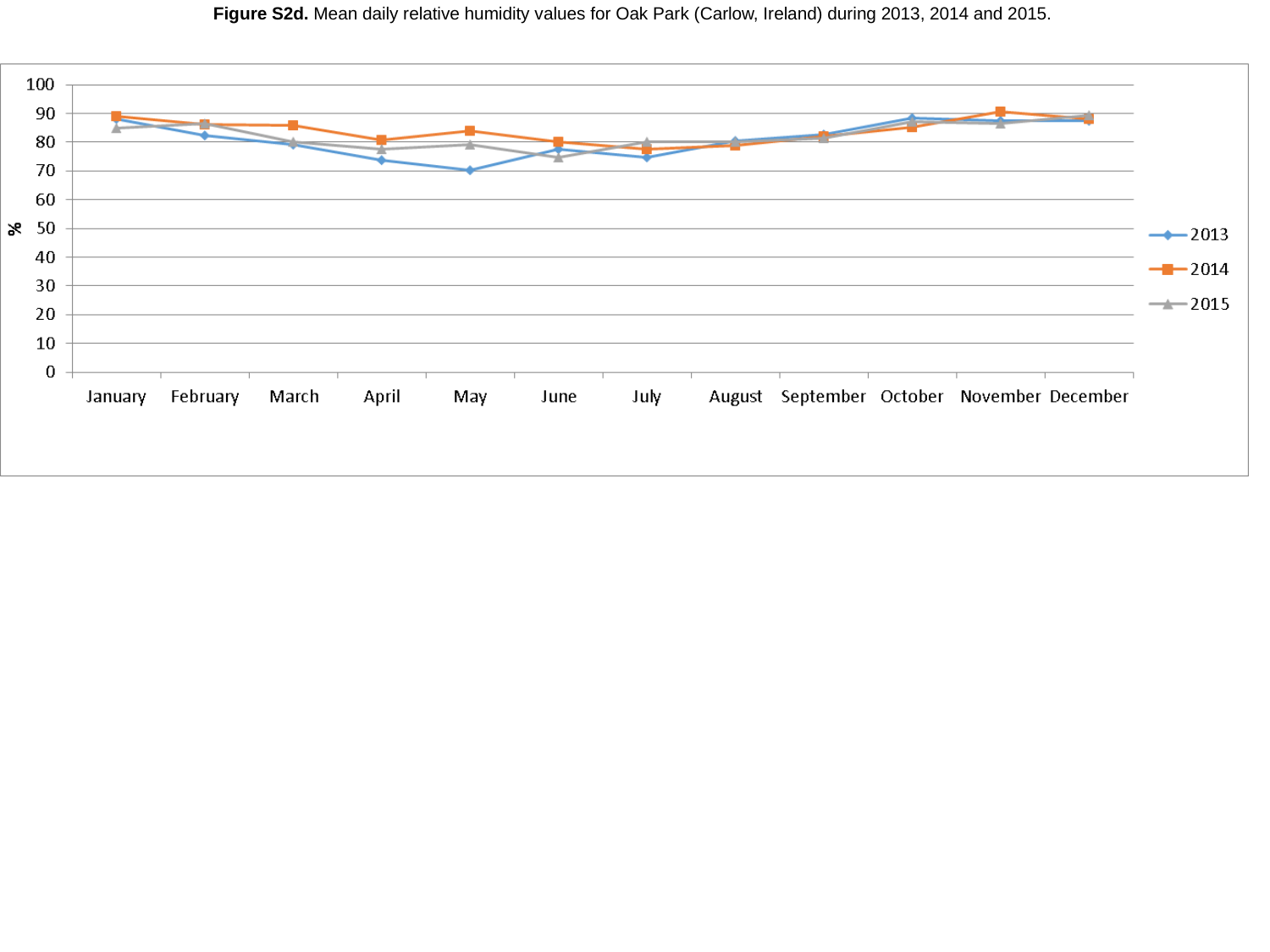

Figure S2d. Mean daily relative humidity values for Oak Park (Carlow, Ireland) during 2013, 2014 and 2015.
