# Supplementary material for: A temporal assessment of nematode community structure and diversity in the rhizosphere of cisgenic Phytophthora infestans-resistant potatoes
Source: BMC Ecol. 2016 Dec 1;16:55. doi: 10.1186/s12898-016-0109-5 (PMC5134073; doi:10.1186/s12898-016-0109-5)
Supplement: Supplementary file 4 — Additional file 4: Figure S3. Uniformity of values across the Shannon Diversity (H) and Shannon Equitability (EH) indices for samples taken from under different potatoes genotypes (Desiree, cisgenic Desiree, Sarpo Mira) treated with different disease management regimes (control, chemical treatment) through the years of 2013, 2014 and 2015 at Oak Park (Carlow, Ireland). [file 12898_2016_109_MOESM4_ESM.pptx]

## Slide 1
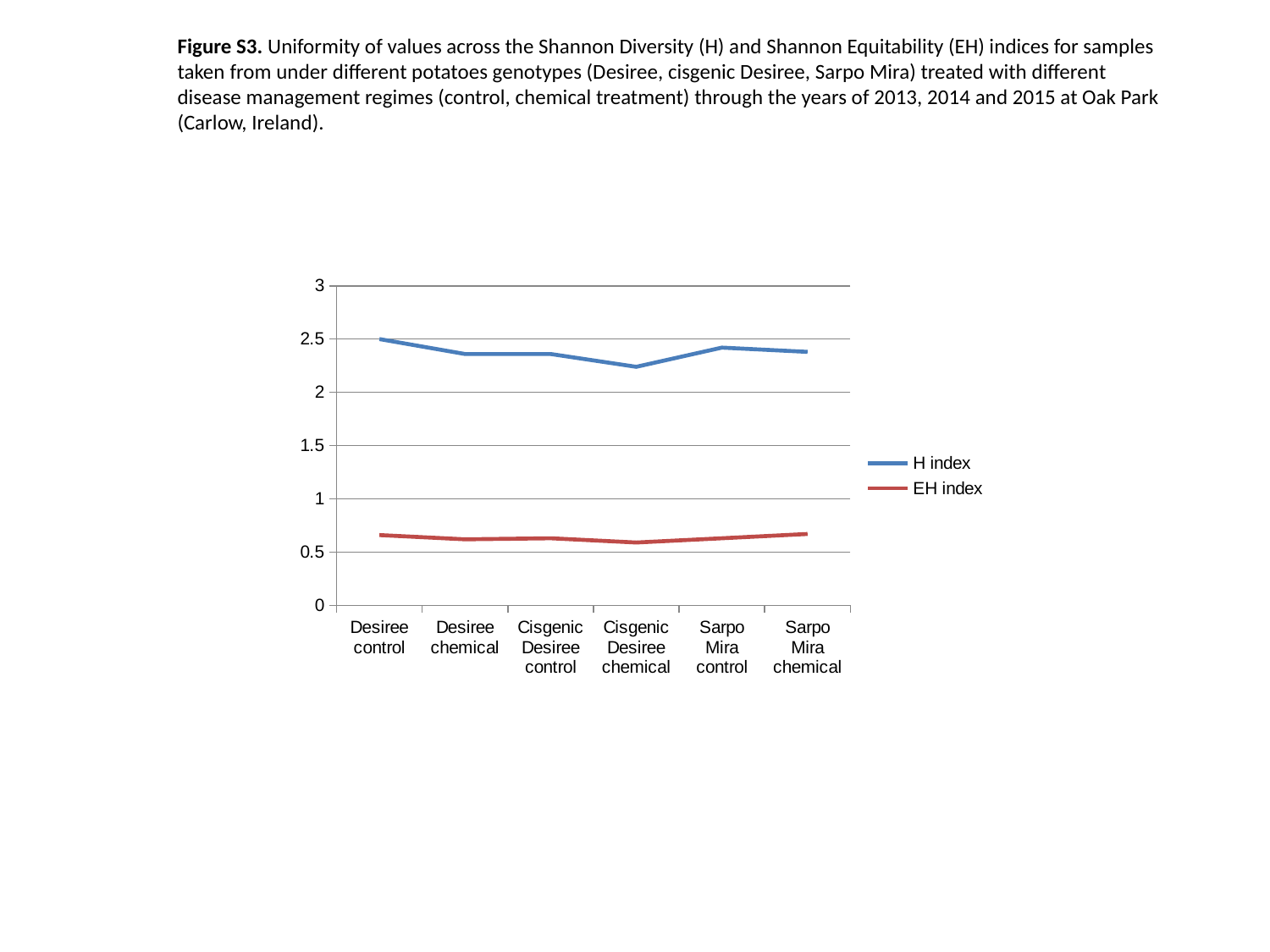

Figure S3. Uniformity of values across the Shannon Diversity (H) and Shannon Equitability (EH) indices for samples taken from under different potatoes genotypes (Desiree, cisgenic Desiree, Sarpo Mira) treated with different disease management regimes (control, chemical treatment) through the years of 2013, 2014 and 2015 at Oak Park (Carlow, Ireland).
### Chart
| Category | H index | EH index |
|---|---|---|
| Desiree control | 2.5 | 0.66 |
| Desiree chemical | 2.36 | 0.62 |
| Cisgenic Desiree control | 2.36 | 0.63 |
| Cisgenic Desiree chemical | 2.24 | 0.59 |
| Sarpo Mira control | 2.42 | 0.63 |
| Sarpo Mira chemical | 2.38 | 0.67 |
